# Modelling of malaria risk, rates, and trends: A spatiotemporal approach for identifying and targeting sub-national areas of high and low burden

**DOI:** 10.1371/journal.pcbi.1008669

**Published:** 2021-03-01

**Authors:** Jailos Lubinda, Yaxin Bi, Busiku Hamainza, Ubydul Haque, Adrian J. Moore

**Affiliations:** 1 School of Geography and Environmental Sciences, Ulster University, Coleraine, United Kingdom; 2 School of Computing, Engineering and Intelligent Systems, Ulster University, Londonderry, United Kingdom; 3 School of Computing, Ulster University, Newtownabbey, United Kingdom; 4 Ministry of Health, National Malaria Elimination Centre, Lusaka, Zambia; 5 Department of Biostatistics and Epidemiology, University of North Texas Health Science Centre, Fort Worth, Texas, United States of America; 6 Department of Geography, University of Florida, Gainesville, Florida, United States of America; 7 Emerging Pathogens Institute, University of Florida, Gainesville, Florida, United States of America; York University, CANADA

## Abstract

While mortality from malaria continues to decline globally, incidence rates in many countries are rising. Within countries, spatial and temporal patterns of malaria vary across communities due to many different physical and social environmental factors. To identify those areas most suitable for malaria elimination or targeted control interventions, we used Bayesian models to estimate the spatiotemporal variation of malaria risk, rates, and trends to determine areas of high or low malaria burden compared to their geographical neighbours. We present a methodology using Bayesian hierarchical models with a Markov Chain Monte Carlo (MCMC) based inference to fit a generalised linear mixed model with a conditional autoregressive structure. We modelled clusters of similar spatiotemporal trends in malaria risk, using trend functions with constrained shapes and visualised high and low burden districts using a multi-criterion index derived by combining spatiotemporal risk, rates and trends of districts in Zambia. Our results indicate that over 3 million people in Zambia live in high-burden districts with either high mortality burden or high incidence burden coupled with an increasing trend over 16 years (2000 to 2015) for all age, under-five and over-five cohorts. Approximately 1.6 million people live in high-incidence burden areas alone. Using our method, we have developed a platform that can enable malaria programs in countries like Zambia to target those high-burden areas with intensive control measures while at the same time pursue malaria elimination efforts in all other areas. Our method enhances conventional approaches and measures to identify those districts which had higher rates and increasing trends and risk. This study provides a method and a means that can help policy makers evaluate intervention impact over time and adopt appropriate geographically targeted strategies that address the issues of both high-burden areas, through intensive control approaches, and low-burden areas, via specific elimination programs.

## Author summary

The WHO Global Technical Strategy for malaria has set an ambitious goal to achieve a global reduction in malaria incidence and mortality rates by 90% and attain elimination in at least 35 countries by the year 2030. Malaria intervention choices and strategies for control and elimination still largely depend on the clear identification and understanding of prevailing variations in local malaria transmission levels. Having reliable and robust methods of selecting and classifying areas as either high or low burden is critical in determining the basis for, and evaluation of, optimal malaria control and elimination strategies at national and sub-national levels. Our study presents a novel statistical method for modelling a combination of malaria risk, rates and trends over time (2000–2015) and space (sub-national level). This approach can be used in malaria endemic countries to help policy makers design, implement and evaluate cost-effective, geographically targeted intervention programmes. These programmes include intensive malaria control approaches in high-burden areas and leveraging malaria elimination strategies in low-burden areas.

## Introduction

Malaria transmission trends and risk of infection are usually heterogeneous in time and space. The ability to detect common spatial and temporal variations of malaria burden in sub-national settings is of great interest and a considerable challenge to malariologists and public health experts in endemic countries.

The global decline of malaria incidence rates has stalled, or the rate of reduction slowed in some countries, particularly sub-Saharan Africa [1]. The 2017 and 2018 World Malaria Reports highlight this stagnation [1–3] and have led to the World Health Organisation’s (WHO) launch of a new country-focused approach known as the “*high-burden to high-impact”* malaria response. They also call for the development of novel methods to address the problem [4,5].

Despite the continued fight against high malaria endemicity for the last half-century, Zambia is among those sub-Saharan countries affected by the reported stagnation in malaria progress [6,7]. With a massive scale-up in interventions [7–10] in the last decade, Zambia achieved considerable progress, resulting in a move away from control targets to elimination aspirations [11]. Zambia embraced the currently renewed global interest for malaria elimination, and strategically positioned itself within a regional and global malaria eradication context.

However, Zambia’s geographical location complicates its malaria control status vis-à-vis its elimination aims. For example, the country’s northern and south-eastern neighbours (Angola, Congo DR, Tanzania, Malawi, and Mozambique) are often among the WHO’s list of *highest-burden countries* [1,2]. In contrast, some of its southern neighbours are regional frontline target countries in the E-2020 malaria elimination programme [12]. Similarly, this northern vs southern epidemiological contrast is manifest sub-nationally as, generally, Zambia’s northern regions have high malaria infections while the southern regions experience the opposite [13,14]. Uncertainty in progress both regionally [12] and nationally [15] has not prevented Zambia from moving forward with its aim to eliminate malaria.

In the past, countries have generally embarked on nationwide elimination efforts or intensifying control in low-burden and high burden areas, respectively. Traditionally delineating these areas was logically based on incidence alone. As elimination and control are becoming a focal problem of subnational importance, malaria programs now have to deal with the challenge of accurately delineating areas to pursue elimination and those in which to intensify control strategies, over and above the challenges posed by border areas.

In order to ascertain the robustness of methods used for selecting these areas of a high or low burden to inform optimal control or elimination strategies, and as a measure of progress towards country elimination targets, scholars have started thinking of better or more robust alternatives. Kitojo et al. recently compared multiple data sources such as the use of malaria tests from antenatal care against population-wide prevalence surveys in children under five years of age to evaluate them as a measure for malaria trends and progress towards Tanzania’s elimination at subnational levels [16]. Routlege et al. used individual-level malaria cases for geostatistical estimates of spatio-temporal transmission to predict the timeline to elimination or the imminent risk of resurgence in China [17], while Amratia et al. used a combination of serology data, case tracing, and case reports in Haiti [18] to comprehensively capture the transmission landscape.

These studies cite the inadequacy of incidence or prevalence as a single metric, and their methods provide alternatives for multi-metric approaches using multiple data sources besides routine data. However, most endemic countries like Zambia have sufficient routinely collected data but limited population-based survey alternatives. We thus provide an alternative multi-metric approach using a single data source by combining three different measures to better understand and guide the classification of malaria burden and help monitor progress towards malaria control and elimination goals.

While malaria incidence or prevalence rate is a good indicator of how many people need treatment, it only offers a snapshot of infections at a given time point, while missing other important underlying factors such as asymptomatic malaria, and differences in care seeking behaviour. Travel and human movement remain key to malaria elimination, especially in low transmission settings, and any local reductions in prevalence are unlikely to persist if surrounding areas maintain much higher prevalence. Similarly, targeting interventions towards outliers with unusually high levels of malaria burden surrounded by low transmission areas, even after accounting for spatial trends, are likely to be more sustainable in the longer term.

While the logic and justification for targeting high burden areas using incidence alone is sound and deep-rooted in decades of use, the challenge is that low-incidence areas with increasing malaria may still be ignored if incidence is the only defining measure. Ignoring such areas with low but increasing malaria incidence (as a low priority) can compound problems later if these areas progress to moderate or even high incidence status. Considering the trend, however, captures not only the stability in spatial and temporal patterns but also gives an additional perspective in areas where elimination efforts may be ongoing or planned.

In Zambia’s approach, elimination is targeted explicitly in subnational areas where the disease exhibits low incidence while control measures are maintained and implemented in the rest of the country [14]. With insufficient levels of funding for malaria control, the *“High burden to high impact”* approach could help reinvigorate the fight against malaria [4] through the more focused and strategic use of evidence-based decision making that can deploy the most effective malaria control tools in areas where they can have maximum impact. The approach presented in this paper supports the identification and targeting of high-burden areas. It also facilitates the optimisation and prioritisation of locally owned country-led health strategies and priorities to achieve their impact maximisation. We add to the literature advocating that, while accepting disease incidence as the primary basis for making decisions on the choice of areas for control or intervention measures, decision making on this basis alone can be optimised and enhanced without incurring any additional data collection costs. We also highlight the ability of our method to define and measure high or low burden areas in line with the *high-burden high-impact* strategy in order to optimise the delivery of control interventions and tools [4].

Identifying the precise quantity and location of the highest-burden areas could help programs focus their limited resources by targeting such areas for further investigations, treatment, prevention efforts, and relevant media campaigns. For example, cost-prohibitive strategies such as mass drug administration (MDA) become feasible for every individual in a small-targeted community hotspot but are not feasible for population-wide application. For instance, Zambia has mostly used targeted indoor residual spray (IRS) to enhance and supplement universal insecticide-treated bed net (ITN) coverage [7]; hence accurate classification is essential in order to ensure the correct application of interventions in true areas of need. Targeting intervention efforts to those places with the highest disease burden relative to surrounding areas is essential because most malaria hot spots are in themselves risks and a source of malaria infections for surrounding areas. Targeting these would help generate a ripple effect that can significantly reduce transmission rates and risk across the recipient areas.

In this study, we investigated the spatiotemporal malaria risk, rate, and trends of all 72 districts in Zambia between 2000 and 2015 using the following process: i) estimate the relative risk and rates of malaria for each district for all ages, under-fives and over-fives, ii) model overall spatial clustering and any related temporal trends and iii) apply a rigorous, but reasonably straightforward, matrix to identify and visualise high burden malaria districts to help inform and support national control and elimination targets. This approach supports and addresses the call for the targeted control or elimination of malaria based on delineated sub-national zones defined by high-burden clusters of risk, rate, and trend.

## Methodology

### Ethics Statement

The National Health Research Authority authorised the study. Study protocols and data requested was reviewed and approved by Ulster University Research Governance (Ref: 17/0049) and the Zambian ERES Converge Institutional Review Board (Ref: 2017-Sept-011).

**Box 1. Nomenclature for equations used**

**Table:** 

*ϕ*	Random effects	**D** = *d*_*tj*_	Temporal neighbourhood matrix
ρs,ρT	Dependence parameters	$T^{2}Q(W,\rho s{)}^{-1}$	Variance
$T_{t}^{2}$	Temporary-varying variance parameter	ρT	Temporal autoregressive parameter
*δ*	Overall temporal trend	*f*_*s*_(*t*|*γ*_*s*_)	Spatial trend
***W***	Adjacency matrix	*k*	Spatial unit
*ω*_*kj*_	Spatial closeness of areal units	*ω*_*κ*_	Binary indicator where *ω*_*κs*_ = 1
*ψ*	Latent component	***λ***	Region-wide probability
t	Timepoint	***α***	Priori distribution

### Study area

Zambia is a landlocked country in South Central Africa, neighbouring eight other malaria-endemic countries [7,19], three of which, represent the frontline region-specific Elimination8 (E8) and E-2020 malaria elimination countries [20]. Zambia’s geographic location creates a heterogeneous and complex malaria transmission landscape that is suitable for tailored micro-geographic intervention approaches.

### Spatial, population, and malaria data

District populations in the period from 2000 to 2015, were estimated using intercensal and postcensal exponential population growth model information from the Central Statistics Office (CSO) reports from 2000 and 2010 [21]. Post census population estimates and age groups of under-five and over-five-year-olds were obtained from the 2013 CSO report [19]. The derived estimates formed the basis for calculations of malariometric indices such as mortality and morbidity rates by age groups.

The inclusion of two age groups (<5 and ≥5) in this study was influenced by two main factors. Firstly, the data was primarily made available in the three age categories of <1, 1–4, and ≥5 years. We grouped the first two categories into one 0–4 years to be consistent with the national and global malaria priorities and reporting. Secondly, due to the high susceptibility risk, vulnerability, and severity of exposure to malaria infection or disease among under-five children, it has been a focus and priority in the last decade to track progress in under 5 children malaria mortality and incidence. This has since defined the subsequent focus on the under-fives as reported in all Malaria Indicator Surveys (MIS), Demographic Health Surveys (DHS), and World Malaria Reports (WMRs) [22,23].

We obtained malaria epidemiological data through the Ministry of Health (MoH). Clinical and microscopy-confirmed malaria deaths and cases disaggregated by age groups were reported quarterly before 2008. With the countrywide introduction of rapid diagnostic tests (RDTs) between 2008 and 2011 [9,10,24–27], clinical and confirmed cases were reported separately and monthly [28]. In order to retain the usability of the full dataset from 2000 to 2015, we analysed our data annually and maintained the 72 original districts, using a combination of both confirmed cases and unconfirmed malaria cases. We computed malaria standardised incidence ratios (SIR) per 1000, and standardised mortality ratios (SMR) per 10,000 people using a simple formula: SIR = (Observed Cases/Expected Cases) & SMR = (Observed deaths/Expected deaths).

The data’s completeness reporting between 2000 and 2008 was not available at district or health facility level. Instead, national averages were used from WHO’s WMR reports which were consistently high over that period, with a median = 87%, mode = 87%, mean = 88.3%, and SD = 2.97%. Completeness data, however, was available at health facility-level and was utilised to more accurately adjust the district-level data between 2009 and 2015. Information on the missingness of data was only available at the district level. Thus, although missingness was dealt with at the district level, it is highly likely that any variations at the facility level will not be detected. However, missingness at district level stood at 3.4% in deaths among those aged 5 years and over, 2.7% in under 5 deaths, and only 0.1% for reported morbidity.

Among the suspected causes of missingness include a lack of adequate training for the person reporting, understaffing, disease burden, levels of education or simply human error in the collection and aggregation of data. An important consideration in this study was the possibility of spatial bias in completeness/missingness of data. We used the Getis-Ord Gi* Spatial Statistic to test for spatial bias in data incompleteness (as a proxy for missingness) and found that incompleteness was random with a Z-score = 0.049 and p-value = 0.96. On this basis, we assumed that any missingness was completely at random (MCAR).

We used Random Forest to impute the 5% of missing values in the data. From missing values among malaria deaths alone, the normalised mean squared error (NMSE) often used to represent error derived from imputing missing values was 0.22 (22%), while it was 0.072 (7%) for missing case values and 0.094 (9%) overall for the whole dataset.

We, however, did not adjust them for confirmation rates by use of Test Positivity Rate (TPR) because TPR was neither available nor collected between 2000 and 2008. In most instances, information on testing was not available at facility or district-level between 2009 and 2015 either.

### Spatio-temporal modelling

We used a Conditional Autoregressive (CAR) prior method. The CAR method incorporates spatiotemporal generalised linear mixed models for unique areas with inference in a Bayesian environment using Markov Chain Monte Carlo (MCMC) simulations [29–32]. Our model choice is based on its robustness and capability to estimate the effects of risk factors on response variables such as incidence and mortality [33]. We used the models for identifying clusters of neighbouring districts [34] that display a repeated high risk [35] of malaria compared with other adjacent areas. These models account for spatiotemporal variations within the same environment, mainly when using the CARBayesST R package [33,36,37]. Malaria data counts are observed within districts with an assumption that the data has an independent distribution using a Poison model. The model hierarchy defined and specified within its prior distributions would accommodate for any spatial correlations within the data. *(See S1 Appendix).*

The two main models performed in this study included generalised linear mixed models of various forms. The first generates spatiotemporal patterns in the mean response with a general temporal effect but separate independent spatial effects for each year [38]. This model is defined by Equation (1):

$$\psi =\phi_{kt}+\delta_{t},$$

where

$$\phi_{kt}|\phi_{-kt},W∼N\left(\frac{\rho s,\sum_{j=1}^{K}\omega_{kj}\phi_{jt}}{\rho \sum_{j=1}^{K}\omega_{kj}+1-\rho },\frac{T_{t}^{2}}{\rho \sum_{j=1}^{K}\omega_{kj}+1-\rho s}\right),$$

$$\delta_{t}|\delta_{-t},D∼N\left(\frac{\rho \tau ,\sum_{j=1}^{N}d_{tj}\delta_{j}}{\rho \tau \sum_{j=1}^{N}d_{tj}+1-\rho \tau },\frac{T_{t}^{2}}{\rho \tau \sum_{j=1}^{N}d_{tj}+1-\rho \tau }\right),$$ (1)

$$T_{1}^{2},\ldots ,T_{N}^{2},T_{T}^{2},∼Inverse-Gamma(a,b),$$

ρs,ρT∼Uniform(0,1).

We used this model to show the common overall spatial effects for all periods, a common temporal trend, and independent space-time interactions.

The second model is used for districts based on their temporal trends in the risk of malaria infection or death, with trend functions optimised by fixed parametric forms or constrained shapes [35]. We used the model’s effects to follow a multivariate autoregressive process with order 1, using the Equation [2]:

$$\psi =\phi_{kt}+\sum_{s=1}^{S}\omega_{\kappa s}\;f_{s}(t|\gamma_{s}),$$ (2)

$$\phi_{k}|\phi_{-k}∼N\left(\frac{\rho \sum_{j=1}^{K}\omega_{kj}\phi_{j}}{\rho \sum_{j=1}^{K}\omega_{kj}+1-\rho },\frac{T^{2}}{\rho \sum_{j=1}^{K}\omega_{kj}+1-\rho }\right),$$

$$T^{2}∼Inverse-Gamma(a,b),$$

ρs,ρT∼Uniform(0,1).

ωk=(ωk1,…,ωkS)∼Multinomial(1;λ),

$$\lambda =(\lambda_{1},\ldots ,\lambda_{S})∼Dirichlet(\alpha =(\alpha_{1},\ldots ,\alpha_{S})),$$

$$\mathrm{Where}\;\phi_{-k}=\left(\phi 1,\ldots ,\phi_{k-1},\phi_{k+1},\ldots ,\phi K\right).$$

Our model was implemented with 4 MCMC chains and 20000 samples obtained by generating 220 000 samples and removing the first 20 000 as burn-in. We applied thinning on the remaining 200 000 by 10 to reduce the autocorrelation. The outputs from this model include a spatial visualisation (map) with credible intervals, a trend classification probability, a slope of trend change and summaries of the trend outcomes and parameters. However, although we use all these for our interpretation, we do not discuss any of these in the text except the trend visualisation.

Finally, we classified and visualised districts as high-burden or low-burden based on a matrix score using the combined values of relative RIsk, RAtes, and risk Trend (RIRAT) implemented in ArcGIS 10.5 *(See also S1 Appendix).*

## Results

### The spatiotemporal trend of malaria mortality and incidence rates from 2000 to 2015

**Fig 1:**
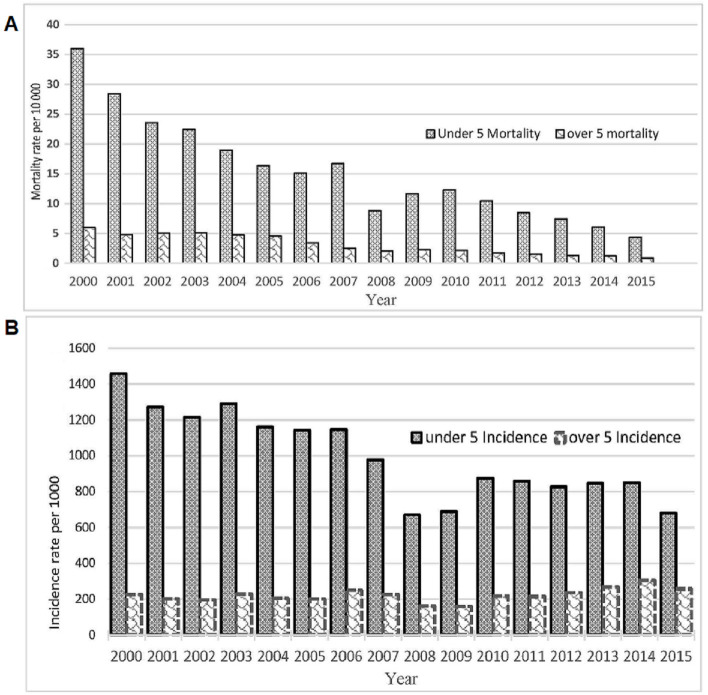
A) Comparative temporal trends in malaria mortality among under 5 and over 5 populations. B) Comparative temporal trends in malaria incidence among under 5 and over 5 populations.

Preliminary analysis of results show temporal progress in the reduction of malaria mortality; however, the trend of malaria incidence remains high. Fig 1A shows a significant decline of about 80% in overall malaria mortality from over 11 500 deaths in 2000 down to near 2300 in 2015. Mortality rates among under-five children showed the most significant decline from 28 down to only 3.3 per 10 000 population at a 95% confidence interval, representing a circa 90% decline. Mortality among the over five population also declined from about 5.9 to 0.58 per 10 000 population.

Fig 1B shows a reduction in incidence rates among under-five children from 1457 to 680 (95% CI) per 1000 population with an average reduction of 44 cases annually. Meanwhile, there was a 14% increase in malaria incidence among the over-fives from 224 to 255 per 1000 population (95% CI).

**Fig 2:**
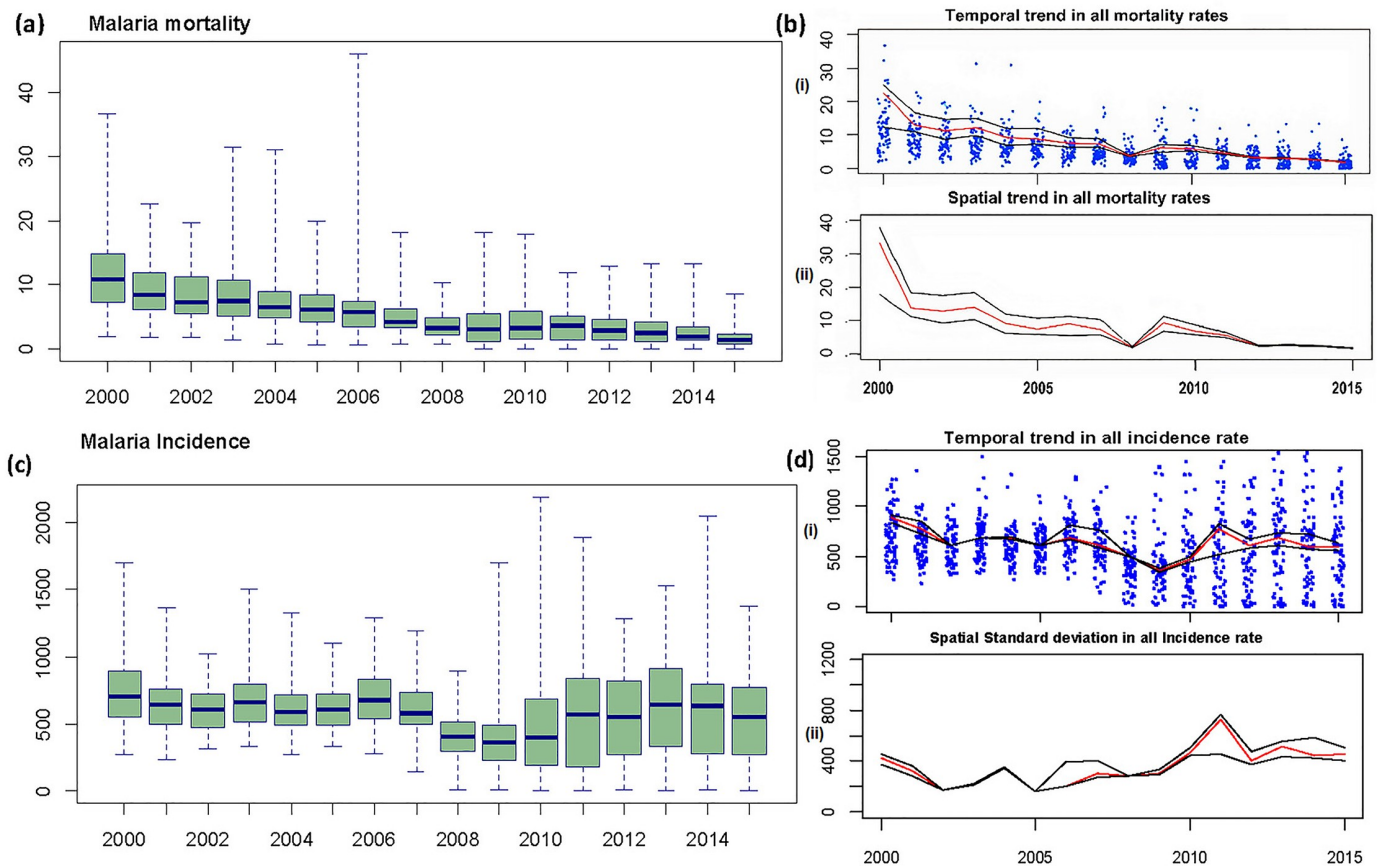
Box plots vs spatio-temporal trends and deviations in transmission. (a & c) are box plots showing temporal mean and inter-quartile ranges of all age malaria mortality and incidence from 2000–2015. (b&d) show the temporal and spatial standard deviations in mortality and incidence rates (Posterior median (red); 95% credible interval (black); each blue dot representing the mortality/incidence rate for each district and year). Temporal mortality decline is associated with increasing spatial homogeneity (a & b), while the incidence trend is more temporally and spatially heterogeneous over the same period. (c & d).

Fig 2 shows the spatiotemporal trends of malaria mortality (2A and 2B) and incidence (2C and 2D) for all ages. Fig 2B and 2D shows temporal trends highlighted by the posterior national median (red) and 95% credible intervals (black) for (i) countrywide mean mortality rates and (ii) the level of spatial standard deviation in mortality and incidence trends. The blue dots are mortality and incidence rates for each district by year. The figures confirm that mortality has declined steadily over the study period with a significant decrease in spatial variance across the 72 districts resulting in a homogenously low risk across the whole country by 2015. In contrast, incidence rates have been unstable with a noticeable increase since 2008, along with an increase in spatial variance across the 72 districts.

Although the change in testing and treatment guidelines around 2008/9, with the introduction of RDTs, could have affected some of the observed declines or no change in trends due to expected decreases in diagnosis of malaria, the observed increases in malaria trends should be deemed independent of this effect because the introduction of RDTs would have eliminated most of the excess non-malaria fever cases. This is because malaria case counts pre-2008 were primarily overestimations (comprised of true malaria as well as non-malaria fevers) [25]. Besides, the results presented here still conform to those reported by Zambia’s microscopy slide-based malaria indicator surveys (MIS) from 2006, 2008, 2010, 2012, and 2015, which show a decline in malaria between 2006 and 2008, but a speedy rise from 2008 to 2015. MISs are independent household surveys undertaken to collect national and subnational data from representative samples of respondents. They assess, among other things, the coverage of key malaria interventions, measure malaria-related burden using prevalence testing of parasites, anaemia, case management, and knowledge and community empowerment among children under five years old.

Results from MISs also confirm the inherent consistency in the trend captured in the routinely collected data. This is further validated by the improving quality of HMIS data observed from the declining portion of unconfirmed malaria reported in the HMIS from 55% in 2011 to only 20% in 2015 (*see S1 Appendix and S3 Fig*). However, these observed spatial variances may be a result of factors such as staggered interventions, especially IRS, which is not applied consistently in specific districts but rather targeted to supplement LLINs in very high transmission areas. This means that the chances of having areas sprayed in one year and not another depending on the preceding year’s transmission levels were high. RDT stock-outs [26,39–45] (recorded at ≈ 20% in 2015) or any differences in the adoption of RDT usage by clinicians could just as well cause such spatial variations. We also further observed that the decline of 2008 predates RDTs by 2 years and comes on the backdrop of the removal of health facility user fees that instead should have increased the cases captured and promote a rise rather than a decline.

**Fig 3:**
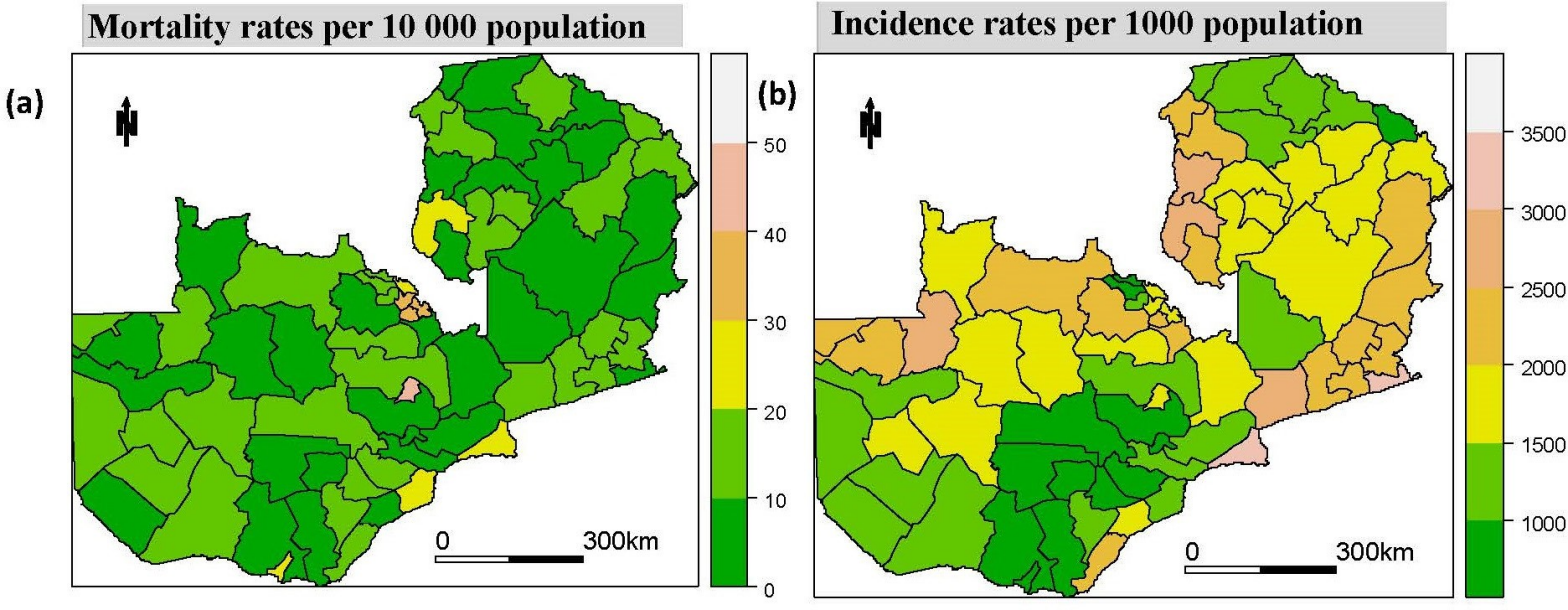
Spatial patterns of malaria mortality and incidence rates, 2000 to 2015. (A) and (B) show the mean spatial pattern of both mortality and incidence rates between the years 2000 and 2015.

It would still be fair to assume that RDT adoption or stock could be an issue due to commodity distribution inefficiencies following this RDT implementation, especially for districts further away from the initial national/central hub. Massive stock-outs especially in further off rural districts, were common before the optimisation of the supply chain as summarised in Vledder [45]. Flaws in the medical supply chain management of commodities and equipment, however, have also been acknowledged in many other studies and reports [26,39–44]. Nonetheless, these persistent stock-outs have significantly reduced in number although they may still have random spatio-temporal effects across the period. Despite the challenges highlighted above, it is worth noting that the documented variability in the availability and application of RDTs and control measures across districts was not consistent through time or space, and therefore cannot explain the long term trends discussed here. The spatial patterns for both mortality and incidence rates can be seen in Fig 3A and 3B.

### Spatial patterns of malaria risk from 2000 to 2015

**Fig 4:**
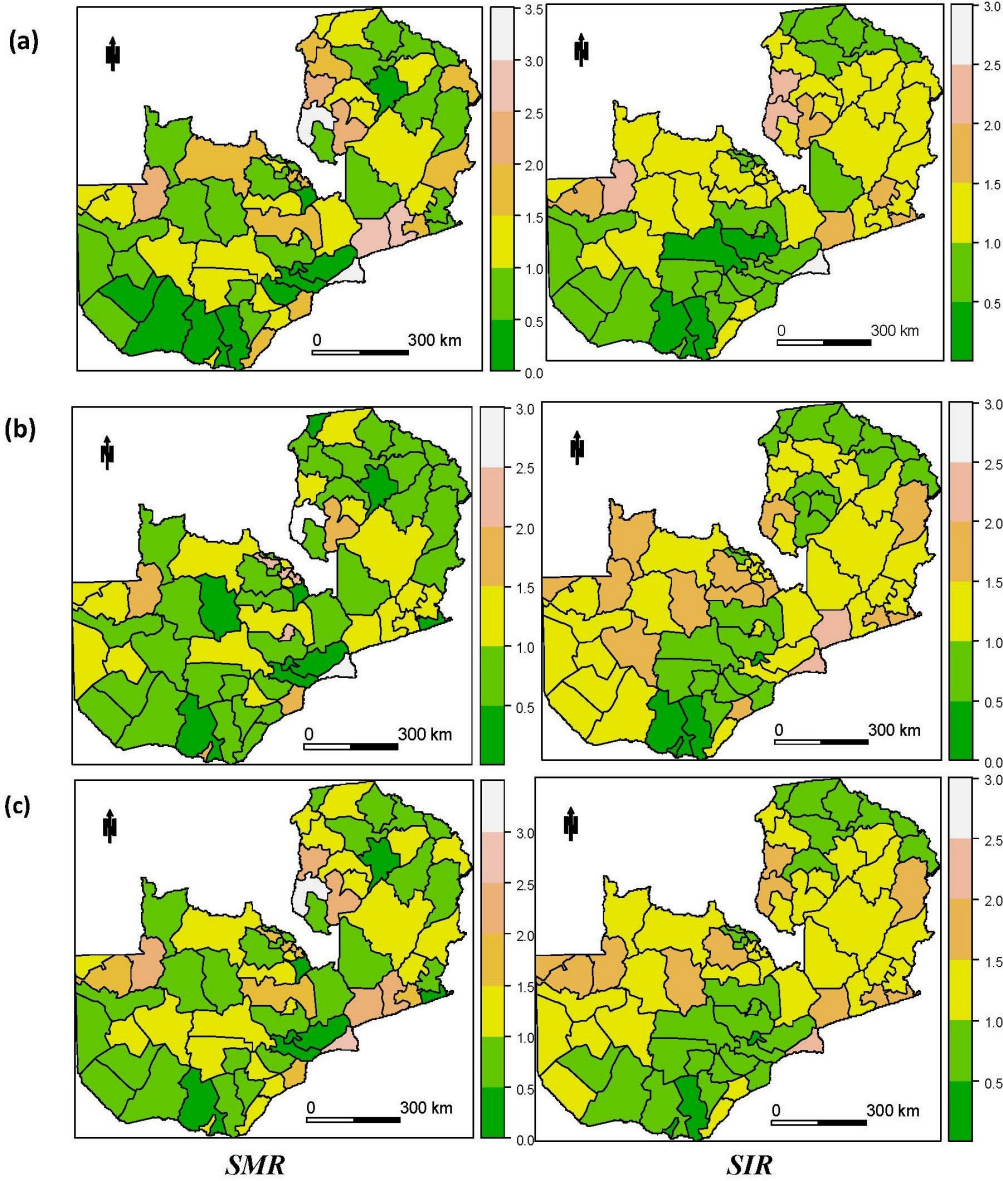
Relative risk of malaria mortality (SMR) and incidence (SIR) among under-fives, five and over and all ages, 2000–2015. (A) = Under-fives, (B) = five and over (C) = all ages.

Fig 4 shows the district level relative standardised mortality risk (SMR) and standardised incidence risk (SIR) for all age, under 5 and over 5 categories. The interpretation of the risk scores is that an SMR/SIR of 1.5 corresponds to a 50% higher risk compared with the countrywide average while an SMR/SIR of 0.9 denotes a 10% lower risk. Based on the calculated SMR, few districts indicate a higher risk of mortality among under-five populations. Notably, some districts in the Eastern and Northern provinces have more than a 250% higher risk of malaria mortality for the under-five age group above the national average, and generally, the Eastern province had the highest risk across the country. The figures also support the temporal trends observed earlier in that the under-fives have a higher risk compared to the over-five age-group. The risk of infections also shows the similar but less extreme variance in spatial patterns with concentrations of low-risk areas in the south and parts of the Central and Northern provinces.

### Spatial Clustering of areas exhibiting similar malaria trends

**Fig 5:**
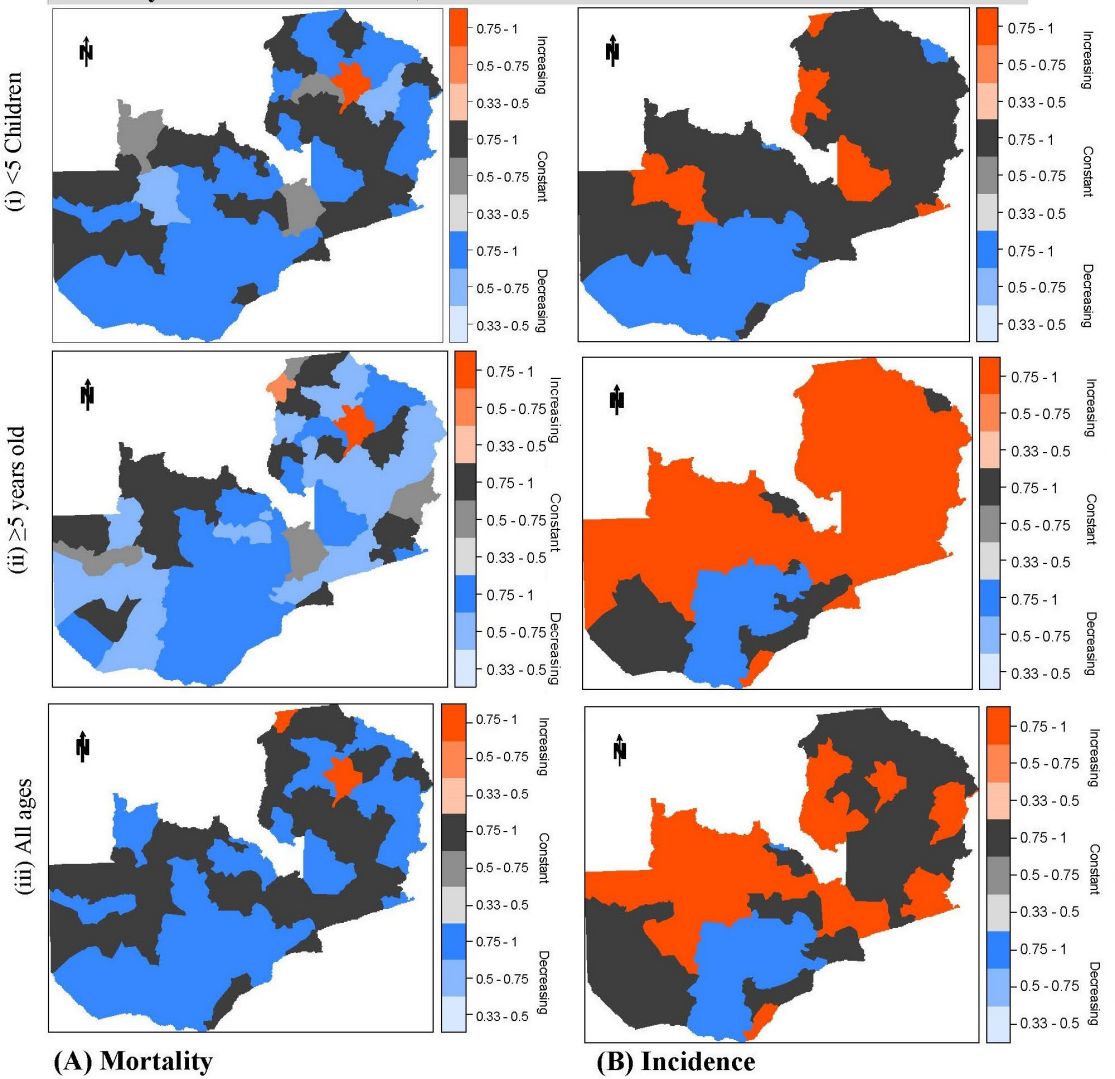
Temporal trend of malaria under-five children and over five age group mortality and incidence trends, 2000–2015. The *red* trend shows *increasing*, the blue trend shows *declining*, and the *black* trend shows *constant/no change*. Classifications are based on the maximum posterior probabilities: the *darker/deeper* the shading, the higher the posterior probability for that trend and vice versa. Fig 5(iii) is also published elsewhere [61].

Fig 5 shows the distribution of district clusters exhibiting similar temporal malaria risk trends. Districts were categorised as having either an increasing trend (red), a constant/no change trend (black) or a decreasing trend (blue) with the darker/deeper the shading, the higher the posterior probability for that trend and vice versa. There was very little to no posterior uncertainty in the under-five mortality and incidence risk classifications assigned to each of the three trends *(increasing*, *constant/no-change*, and *decreasing*). In contrast, minimal uncertainties (*probability = 0*.*5–0*.*75*) are visualised in the *increasing* over-five mortality clusters and *no-change* in all-age clusters.

With regard to mortality trends, in those districts with either a constant or decreasing trend, the pattern of change in trend over time has levelled-off and currently remains steady. However, in those districts where the mortality trend has been increasing (i.e. 7% of districts for under-fives and 32% districts for over fives) the pattern of increase during the 16 year study period has been rising. This would indicate that there is a worsening situation in malaria mortality in those areas, creating a real potential to negatively influence national mortality figures if this situation continues *(See S1 Fig).*

Progress in reducing under-five mortality over the 16-year study period is consistent and evident across risk, rates, and trends while incidence across the three age categories is less consistent and more varied. Only 3% (2) of districts showed an increasing trend in under-five mortality while 71% and 26% experienced a decreasing trend or no-change, respectively (Table 1). For incidence risk in the under-fives, however, there was an increase in 10% of districts (mainly around the northern half and easternmost border region), a decrease in 20% of districts around southernmost areas. In comparison, 45% remain unchanged (clustered mainly around the middle half of the country). The mortality trend among the over-fives is more varied (Table 1) with 3%, 69% & 28% of districts either increasing, decreasing or no-change, respectively with a model classification certainty of 75–100% (Fig 5A [ii]).

**Table 1:** Summary description of malaria mortality and incidence trends (<5 years children, ≥5 years, and all ages combined. **NB** *% represents the proportion of the 72 districts assigned to each trend*, *i*.*e*. *Decrease*, *Increase*, *or No-change*.

	Mortality			Incidence		
Age group	Districts	%	Trend	Districts	%	Trend
under 5	19	26.4%	No change	45	62.5%	No change
	51	70.8%	Decrease	20	27.8%	Decrease
	2	2.8%	Increase	7	9.7%	Increase
over 5	20	27.8%	No change	17	23.6%	No change
	50	69.4%	Decrease	10	13.9%	Decrease
	2	2.8%	Increase	45	62.5%	Increase
overall	31	43.1%	No change	34	47.2%	No change
	39	54.2%	Decrease	13	18.1%	Decrease
	2	2.8%	Increase	25	34.7%	Increase

A large cluster of districts in the southern region has a decreasing trend relative to the rest of the Country (Fig 5B (i-iii). The trend for over-five incidence in Table 1 shows that over half of all districts (62%) are increasing, while only 14% are decreasing and 24% exhibit no-change (all results are statistically significant at a 95% credible interval).

### A classification matrix for determining overall malaria burden

**Fig 6:**
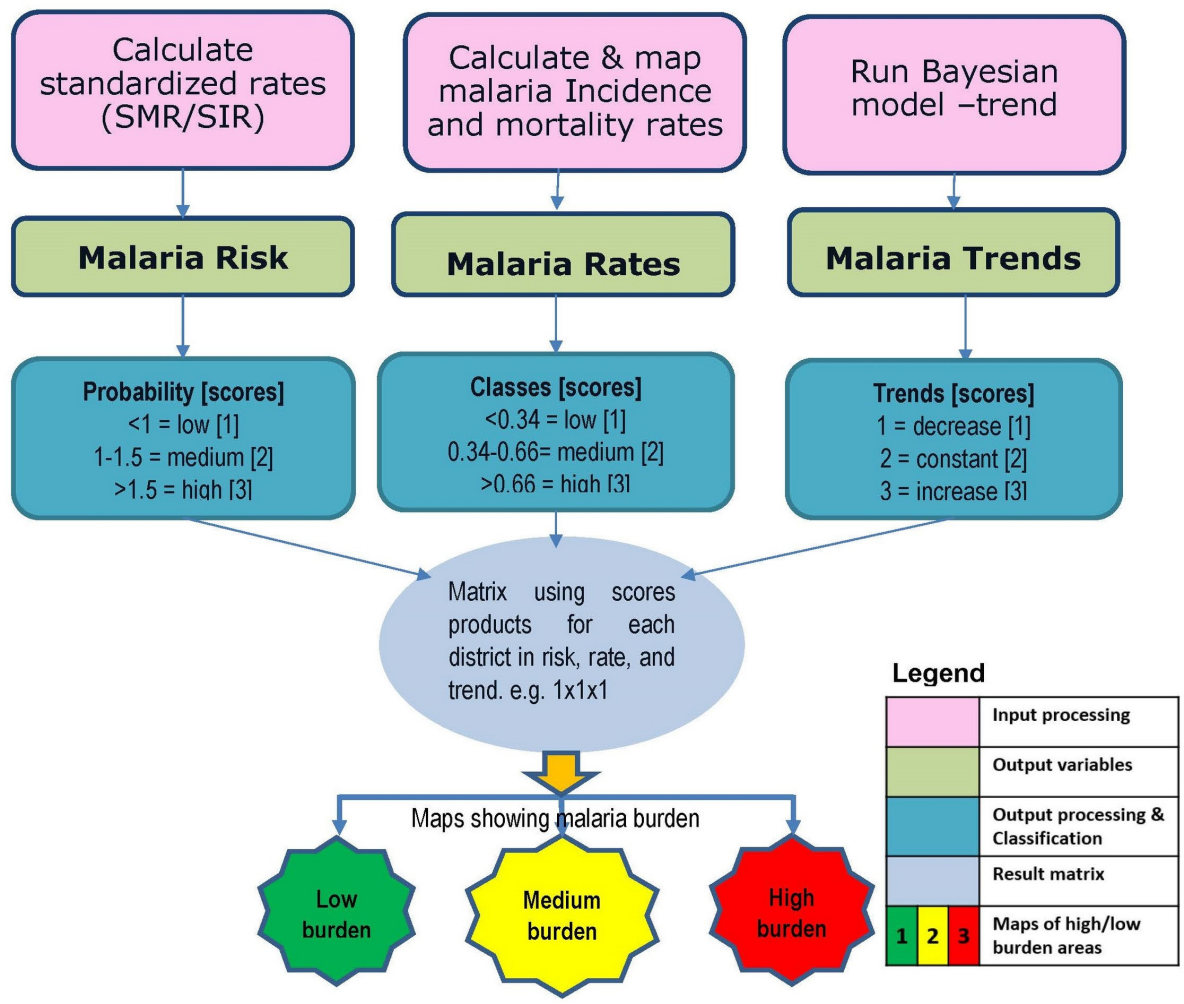
Data preparation and processing to determine areas of highest/lowest burden. Stages of data analysis from initial, intermediate, to final outputs. The classes relate to 1, 2, 3 scores with 1 = low, 2 = medium and 3 = high applied to risk, rates, and trends.

While rate and risk trend clusters show a clear picture of overall district-level classification, i.e. decline, no-change or increase as illustrated in Fig 4 and Table 1, reviewing these trends separately may conceal or mask the overall underlying picture which in turn might undermine the actual implications of these trends for malaria control. For instance, a district with high risk, high rate, and showing no-change in trend could be more alarming compared to a district that has low risk, low rate and no change or an increasing trend. Therefore, we created a matrix of the combined indices for malaria RIsk, RAtes, and Trends (RIRAT) to accurately classify high-burden and low-burden districts (Fig 6) (*See also S1 Appendix*).

**Fig 7:**
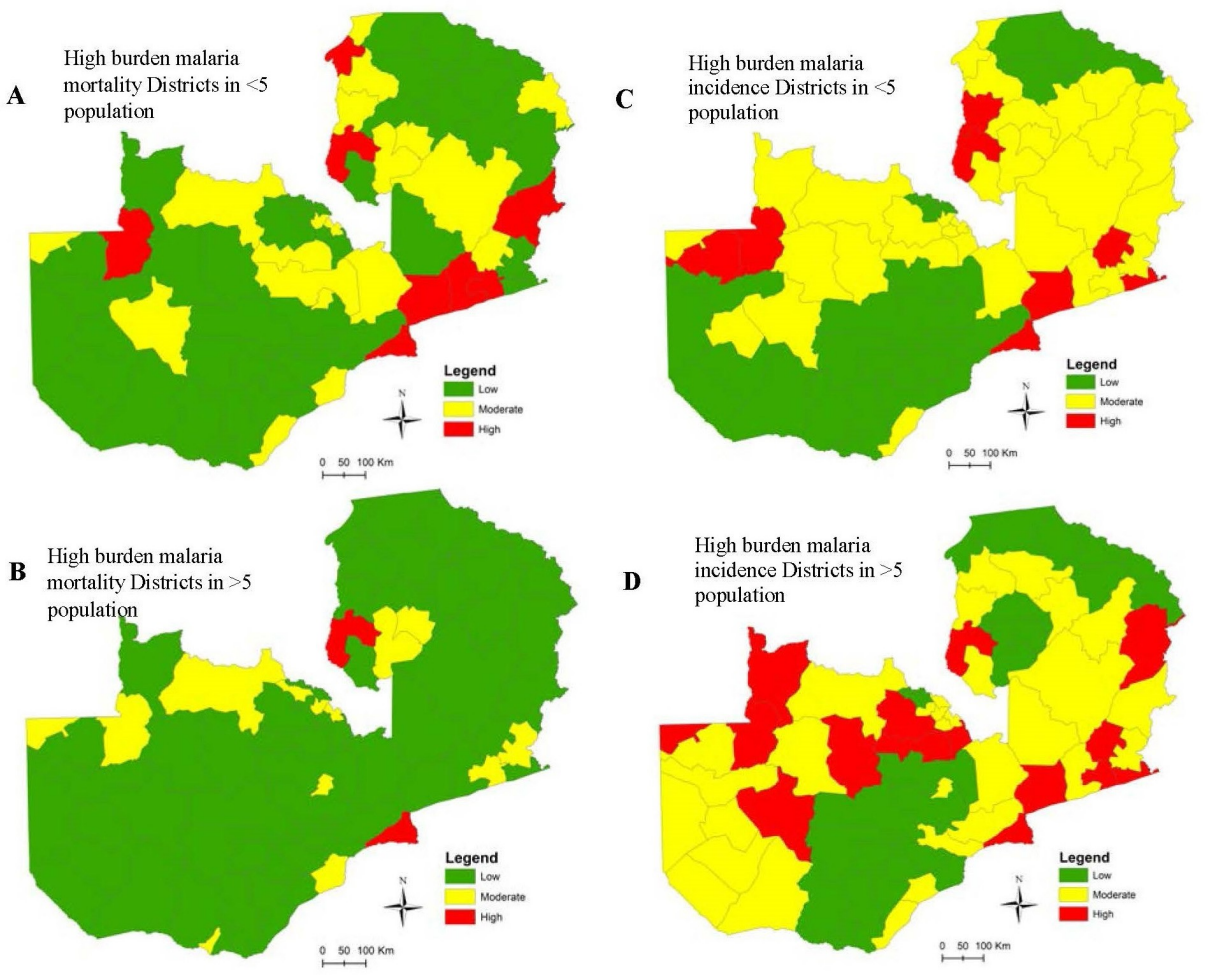
High/low burden malaria mortality (A) & (B) and incidence (C) & (D) districts using matrix scores of risk, rate, and trends.

Fig 7 shows comparative district level maps for mortality and incidence burden for the two age categories on the spectrum of *low (green)*, *medium (yellow)*, and *high (red)* -burden (*See S1 Table for details*). Fig 7A and 7C shows the districts in 2015 (mostly in Eastern and Luapula provinces) with the highest under 5 mortality-burden (8 districts) or highest incidence-burden (8 districts) representing an estimated half a million children in that age cohort. Twelve unique districts were classified with either high-mortality or high-incidence burden while four had both. For the over-fives, 15 districts were identified as high-incidence burden areas representing approximately 2 million people in that age group. Only two districts had both high-mortality and high incidence burden representing about a quarter-million vulnerable people, while an additional 1.5 million people aged over five lived in the 13 districts with a high-incidence burden only.

Derived from the matrix score, more than 3 million people live in districts with generally high incidence risk, high incidence rates and an increasing trend. This population is exposed to at least twice the risk of malaria compared to other areas in the country.

To assess our method further, we observed differences among incidence classification of those aged ≥5 years through a comparison of the derived results using raw rates alone against our method. There was a considerable difference in the proportion of districts classified as high or low burden compared to those identified by our method. For example, only 55% of districts identified as high incidence using raw rates alone were also deemed high-burden using the overall weighted combined method. The differences observed here were because high burden districts were showing an overall increasing trend over the period while the others did not. Similarly, 45% of those districts identified as high in the raw rates dropped into the moderate burden category, and 30% of districts identified as low ended up as moderate-burden districts. The differences observed here highlight the limitations of using raw incidence rates as a basis for identifying and targeting intervention strategies at the subnational level.

## Discussion

Our study findings have important implications for malaria policy in Zambia, and the various intervention approaches used within the country. As shown, in both age groups, it is clear that there has been remarkable progress in mortality reduction but less so in incidence reduction. Both the under-five and the over five age groups experienced a similar rate of reduction in mortality (85% and 90% respectively). However, the under-fives continue to experience approximately five times the incidence rates and at least 2.5 times the mortality burden compared to the over-fives. Without overemphasising the observed declining malaria mortality, the overall results would indicate that more can still be done to further reduce the under-five mortality burden by targeting the highest-burden areas. The benefit of the high precision district-level analysis presented in this study provides an opportunity to move away from the *one-size*-*fits-all* approach, and optimise resource deployment in a more focused, efficient and geographically targeted manner. The findings also demonstrate how a small number of high burden areas can skew the national averages and overshadow the actual progress achieved so far in the country as a whole. This study has provided a means of determining districts with high malaria burden where, if prioritised, targeted malaria control efforts could help maximise impact (*S2 Fig).*

With proposed sub-national elimination approaches soon to be implemented in Zambia, our method, based on an analysis of 16 years of data has identified those areas that are most suitable for malaria elimination *(S2 Fig)*. Our method can be applied to help other countries identify high-burden areas and achieve maximum impact through the appropriate use of tools and interventions efficiently and effectively. This is important when considering the use of expensive interventions such as indoor residual spraying, which requires rounds of minimum spray coverage thresholds of up to 85% [46].

An additional point of particular interest in Zambia is that most high-burden areas comprise districts along the national borders with Angola, Democratic Republic of Congo, Malawi, Tanzania, and Mozambique. All these countries are unequivocal, high-burden malaria-endemic countries that have often been ranked among the top ten high-burden countries in the world. This observation highlights the significance of the need for countries to engage in bilateral and collaborative regional malaria initiatives for successful control along borders. While Zambia is part of the Elimination8 countries cross-border malaria collaborations, this only applies in southern bordering countries. No such formal undertakings are present with Zambia’s northern bordering countries [20]. The patterns and trends presented here reflect Zambia’s geographic location and adjacency with contrasting high-burden and low-burden neighbouring countries and highlight the potential influence and impact of cross-border malaria risk in border districts [47,48]. This method, if carefully applied, could additionally benefit other low resource countries and encourage broader regional collaborations, particularly for targeted cross-border initiatives.

We have presented an empirical but rigorous approach for the identification of high-burden/low-burden malaria incidence and mortality in affected countries. In the case of Zambia, we would propose a review of the current under-five malaria intervention strategies, especially in high-mortality burden districts so that any potential problems or issues can be identified and addressed. We would also recommend more focused ongoing operational research to assess progress and identify specific challenges at the community level [49].

**Fig 8:**
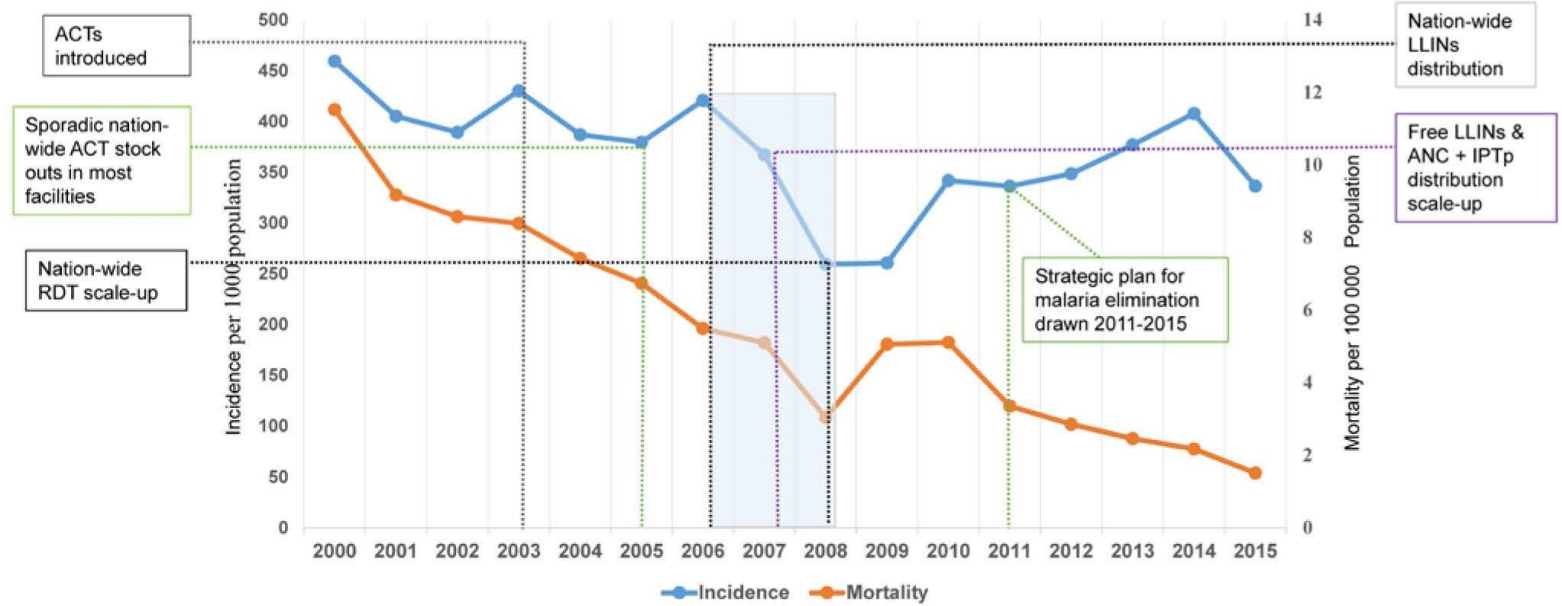
Incidence and mortality trend against major malaria policy changes. Significant malaria policy changes and guidelines, interventions and diagnostics in Zambia 2000–2015. Note that we did not include any changes that were progressive, e.g. IRS. Major policy changes undertaken in Zambia, 2000–2015 (Source of data: Steketee *et al*. 2008, Chanda *et al*., 2013, Redditt *et al*. 2012, Kamuliwo *et al*. *2015*) [2003: Chloroquine (CQ) replaced by artemisinin-lumefantrine (Coartem) as first-line malaria treatment and new diagnosis and treatment guidelines for malaria to reflect drug policy change launched; 2006: Use of Insecticide Treated Nets (ITNs) adopted; 2006–8: Training of additional microscopists, scale-up of RDTs distribution; free distribution of ITNs through antenatal care (ANC) and intermittent preventive treatment (IPT) using sulfadoxine-pyrimethamine (SP); 2011: Consideration for future elimination begins with the alignment of NMCP strategic plan 2011–2015 with the national vision *“a malaria-free Zambia by 2030”*].

While this study focused more on the identification of high burden malaria control areas rather than those most suitable for elimination, the approach still provides sufficient evidence and information that can accurately inform both control and elimination approaches. Our approach provides the information base needed to facilitate further research into the specific factors that might explain within-country differences between regions and age cohorts, including the value and impact of intervention programmes over time. For example, Fig 8 shows the relationship between mortality and incidence trends with significant malaria policy changes and guidelines on interventions and diagnostics in Zambia between 2000 and 2015.

Of interest is the post-2008 trend of increasing incidence rates despite the various intervention strategies. Potential explanations for the observed trends and incidence rate patterns or changing risk patterns include among others low intervention coverage (particularly IRS), insecticide resistance in mosquito nets or low utilisation driven by misuse such as fishing or agriculture fencing or even changing climatic and ecological conditions [31,50]. Our spatio-temporal modelling and the identification of those specific areas where incidence burden and risk is highest provide essential information to support future geographically targeted initiatives. Such initiatives could replace expensive countrywide programmes, thus facilitating more efficient and effective use of scarce resources.

It is recognised that some of the changes in malaria policy, diagnostics, definition, and collection methods during the 16 years may have introduced potential biases in this study. The incorporation of malaria cases by clinical symptoms added some level of non-malarial fever burden, and which could lead to an over-estimation, especially between 2000 and 2008. This bias, however, would be declining in the post-2008 period *(see S1 Appendix)*.

For example, the observed changes in prevalence rates over time may in some parts have been influenced by changes in diagnostic tools or methods used in case reporting rather than representing real changes in malaria incidence. This could be more relevant in rural health facility settings [28] where limited availability of trained human resources still exist. We also note that this study gives a long-term time-series of mean trends, risks and rates up to 2015, and therefore presents conclusions accurate for this period of study. Usage for present and future decision-making would have to be based on an analysis of more recent and relatively short time datasets of 3 to 5 years.

**Fig 9:**
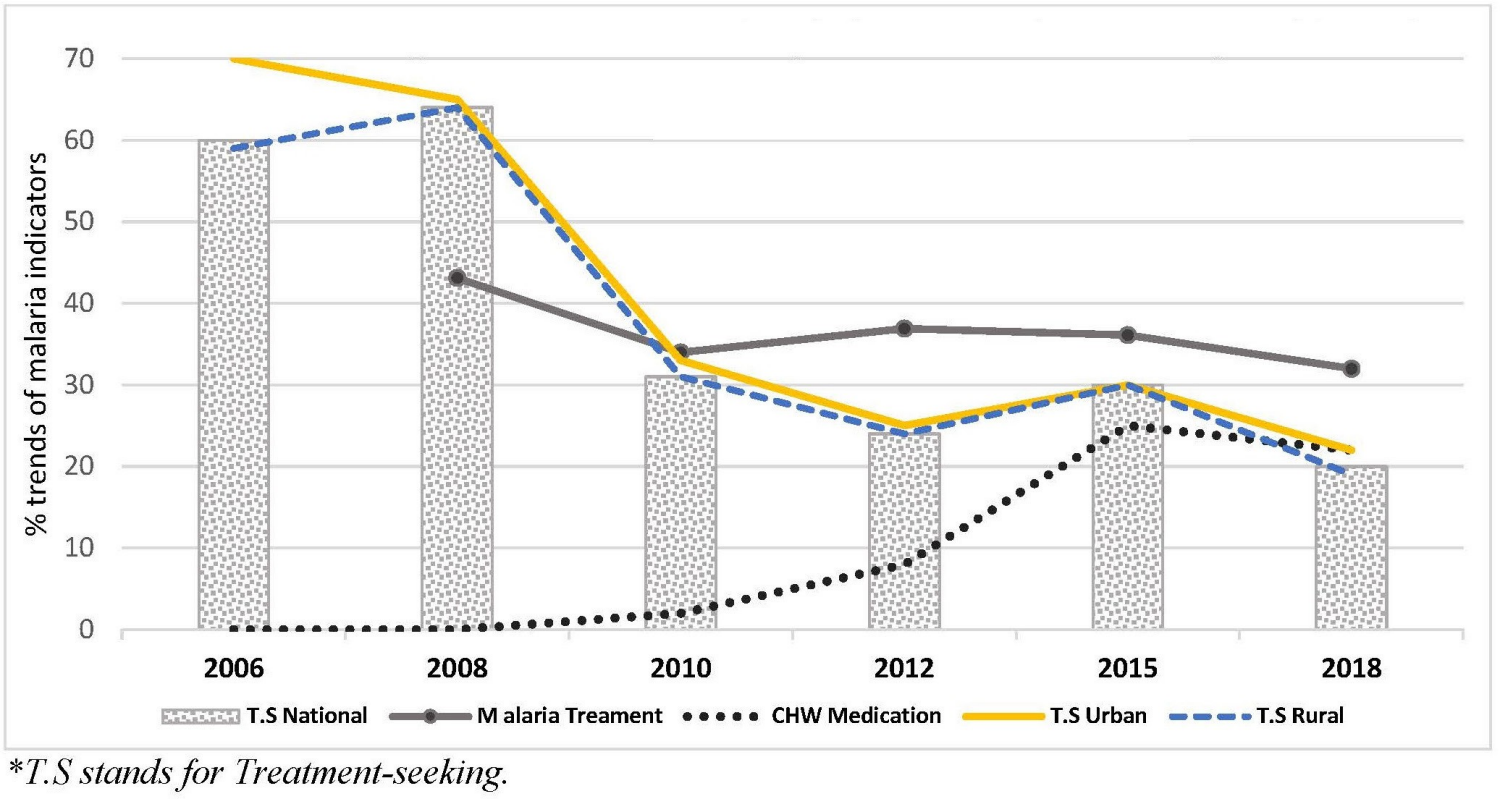
Treatment-Seeking behaviours and Community Health Worker medications in Zambia 2006–2018. *T.*S stands for Treatment-seeking*. ***Data source***: *MIS reports 2006*, *2008*, *2010*, *2015*, *& 2018*.

Progress in health care provision that could have also impacted some of the identified changes in malaria rates may include the creation of new health facilities and the introduction of Community Health Workers (CHWs), particularly since 2010. The assumption is that both these factors could increase access to health care services which in turn would artifactually result in higher treatment-seeking rates and higher case diagnoses. The increase in CHWs is supported by evidence from the MIS 2018 [51] which shows that CHW activity increased during the 2010 to 2015 period of our study. During that period, malaria medication received from CHWs increased across previous MIS surveys from 2% in 2010, 8% in 2012 to 25% in 2015 but down slightly to 22% in 2018 (see Fig 9 *and S4 Fig*). This increase could suggest that CHWs are a possible cause or contributor to the increase in incidence in some areas. However, this argument would be further supported if the increase in CHW treatments was matched with a similar increase in treatment-seeking behaviour. In fact, national data trends show a considerable reduction in treatment-seeking behaviour during the same period in both rural and urban areas (see Fig 9 *and S4 Fig*). So while nationally, there has been an increase in the proportion of patients being treated by CHWs through increased numbers and availability, the actual total proportions of those people seeking treatment has decreased.

This also relates to the assumption that increasing numbers of health facilities may explain some of the increasing incidence trends. Zambia’s primary reason for the construction of new health facilities is often based on population growth with catchment distance and accessibility issues a secondary factor [52–55]. During the period of this study from 2009 to 2015, the number of health facilities reporting in the HMIS increased from 1552 in 2009 to 1869 in 2015. The increase was made up of a combination of government, mission (faith-based), and private providers [52]. During the same period, Zambia’s population increased by 18.5%, while the total number of health facilities increased by 18.7%, and the ratio of health facilities to population remained the same throughout (1.2 health facilities per 10 000 population).

Based on a comparison between population growth and the increase in health facilities, it seems obvious and acceptable to suggest that the construction of new health facilities was primarily driven by population growth. It can also be further argued that the population growth ratio was consistent with the provision of health care facilities per person. Similarly, the incidence rates of malaria would also have stayed the same unless other factors were at play. It would be expected that in either scenario malaria incidence rates would be normalised by the relevant underlying catchment populations and therefore would not likely increase incidence significantly unless the introduction of new facilities resulted in increased treatment-seeking rates. We have seen from Fig 9 that the national treatment-seeking rates in the country actually fell as reported across all the MISs in nearly all provinces and across the rural-urban divide [51,56–60]. In addition, an analysis of national Health Facility data by District from 2009–2015 found a small, but significant, negative correlation (-0.16) between the number of health facilities and incidence rates with over 50% of districts that had an increase in health facility provision actually showing a declining malaria trend and only 33% showing an increase.

In this study, other potential limitations that could have influenced some of the observed results may include: i) the potential presence of unquantifiable effects due to the lack of reliable subnational treatment-seeking rates capable of indicating existing subnational variations if present, ii) uncaptured subclinical malaria which is long known to have a severe impact on transmission, especially in the older age groups due to partial immunity; and iii) the unknown effects of any differences on how quickly RDT use was adopted across the country. These, if present, may affect our conclusions of what the malaria burden in the population actually is (*see S1 Appendix for details on asymptomatic malaria*).

Having said that, the increasing availability (reduced lag) and improvement (in accuracy) of health management information system (HMIS) data presented here provide a much greater opportunity for such data to be used with more confidence in the future. This is particularly true given the more expensive alternatives such as surveys that may not always provide comprehensive longitudinal information and analysis at the times when it is most needed.

## Conclusion

We have presented a method here that augments conventional measures of identifying malaria risk and provides an effective approach for the identification of areas of high and low malaria burden at the sub-national level within countries. By applying a rigorous spatio-temporal approach that uses longitudinal rates, risks and trend clusters, we can help policymakers determine priority areas to deploy scarce resources for high impact control interventions in high-burden areas and elimination strategies in low burden areas.

This easy to implement and replicate methodology will help policy makers and malaria control/elimination program staff in malaria-endemic countries who may not be fully cognisant of or technically skilled in advanced statistical methods (See S1 Code). The novelty of our method is not in the statistical algorithms, which are well-established techniques in their own right, but in the approach of combining the typically independent measures of rates, risk, and trend over time and space that better represent malaria prevalence within a country and are easy to replicate and use at an operational and practical planning level.

We believe that applying this approach could be extremely beneficial to countries embarking on their malaria elimination strategies as part of the global malaria eradication agenda. This could be particularly effective through informed sub-national programs at even finer levels of geographic aggregation, such as health facility catchments, which are well suited for targeted control and elimination strategies.

## Supporting information

S1 Fig Average temporal trends at 95% credible intervals. *Estimated temporal trends and 95% credible intervals in dotted lines*, *arranged to start with under-five (i)*, *over-five (ii) and population-wide (iii)*, *for malaria mortality (A)*, *and malaria infections (B)*.
(TIF)

S2 Fig Maps showing districts with the highest and lowest scores only based on all matrix scores. [Trend u5, Risk u5, Rate u5, Trend o5, Risk o5 & Rate o5]
(TIFF)

S3 Fig HMIS data Quality improvements from 2010–2016. (TIF)

S4 Fig Prompt Treatment-seeking rates, 2006–2018. *Treatment-seeking trends between rural and urban areas of Zambia during the period of study*.
(TIF)

S1 Table Arbitrary combinations of score legends, colours and their resultant score and colour. (XLSX)

S1 Appendix Additional text and explanation of methods. (DOCX)

S1 Code R code used for part of the analysis upon which the manuscript is based. (R)
