# Supplementary figures and images for: Modelling of malaria risk, rates, and trends: A spatiotemporal approach for identifying and targeting sub-national areas of high and low burden
Source: PLoS Comput Biol. 2021 Mar 1;17(3):e1008669. doi: 10.1371/journal.pcbi.1008669 (PMC7951982; doi:10.1371/journal.pcbi.1008669)

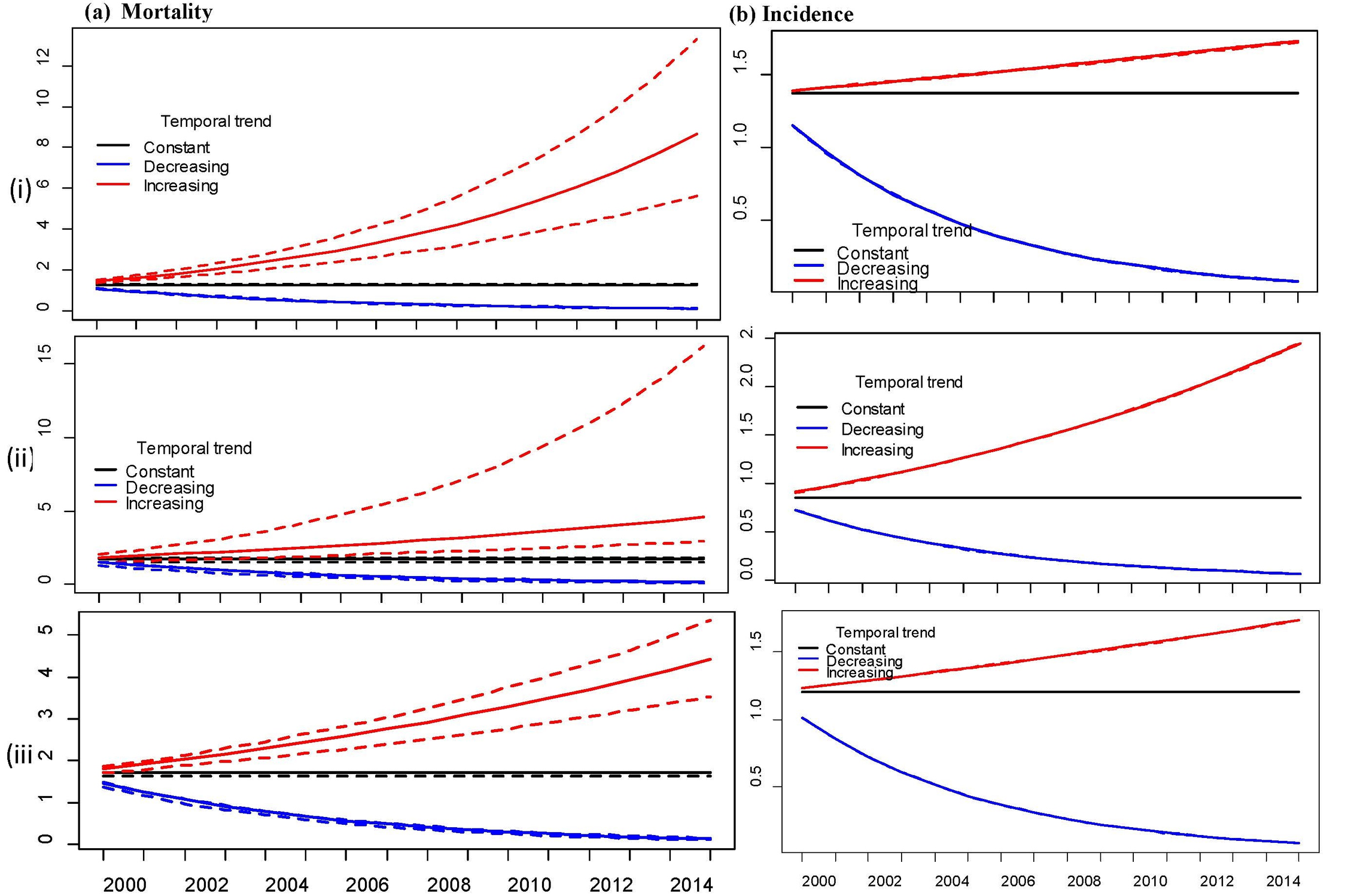

Supplement: S1 Fig — Estimated temporal trends and 95% credible intervals in dotted lines, arranged to start with under-five (i), over-five (ii) and population-wide (iii), for malaria mortality (A), and malaria infections (B). (TIF) [file pcbi.1008669.s001.tif]

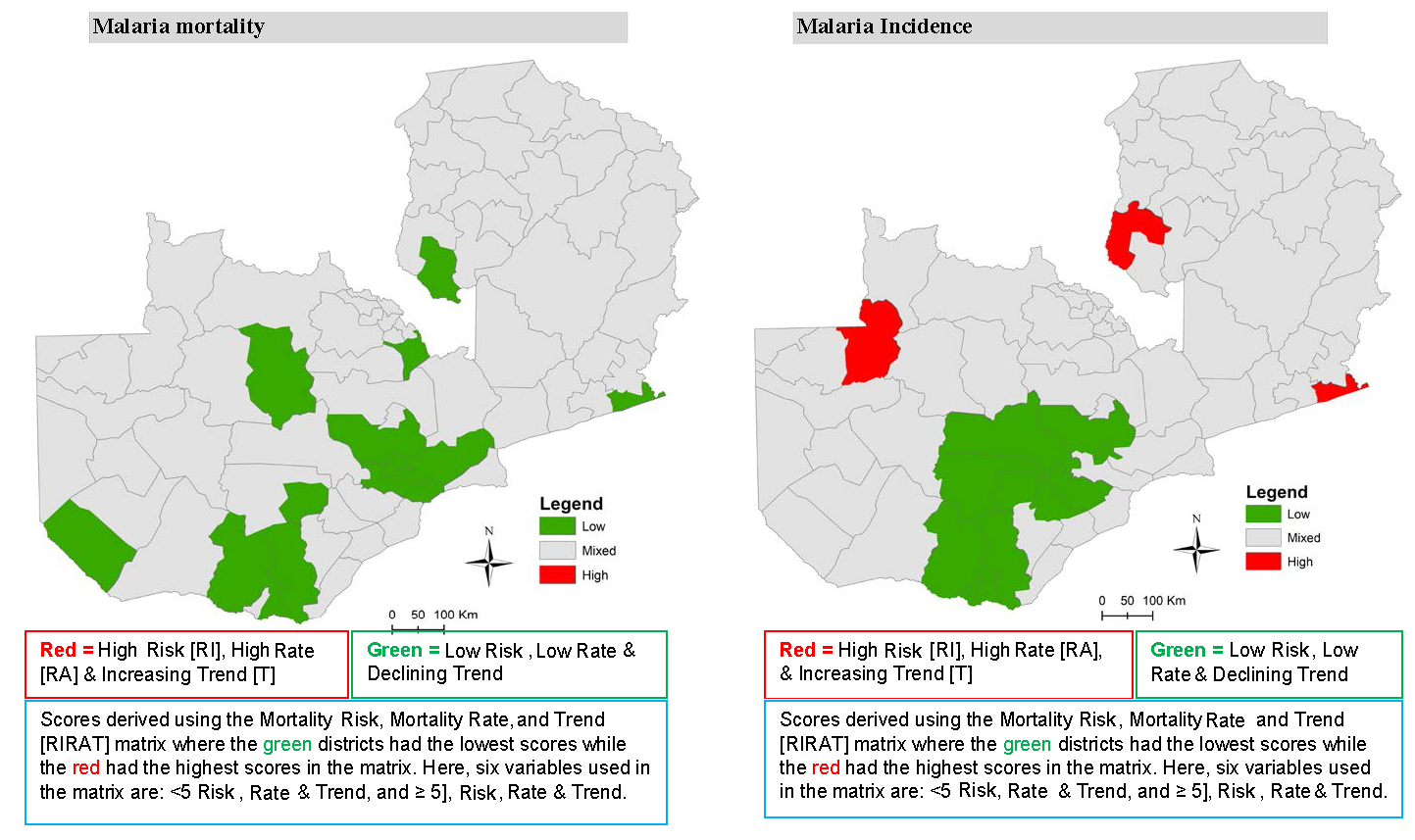

Supplement: S2 Fig — [Trend u5, Risk u5, Rate u5, Trend o5, Risk o5 & Rate o5] (TIFF) [file pcbi.1008669.s002.tiff]

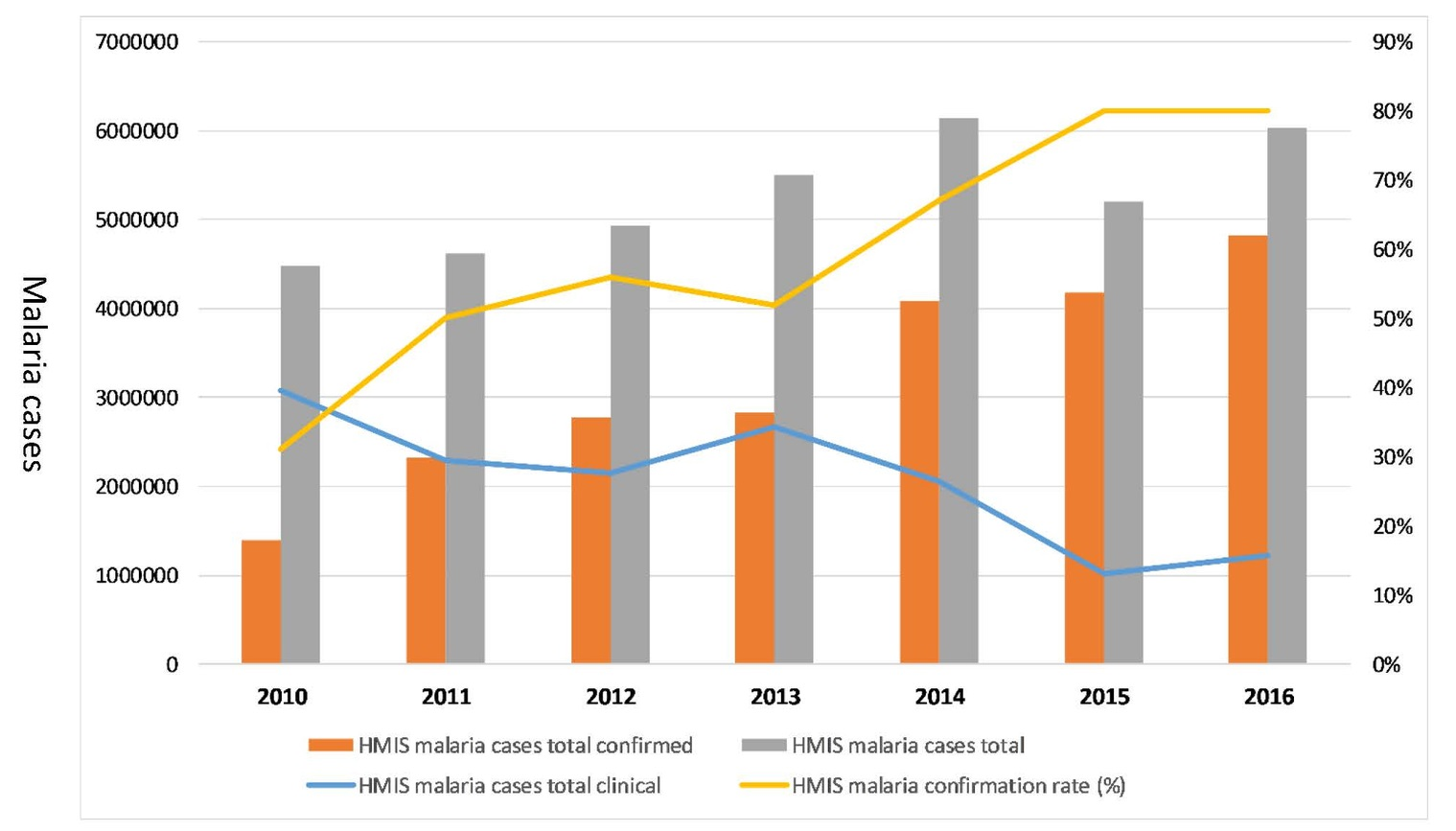

Supplement: S3 Fig — (TIF) [file pcbi.1008669.s003.tif]

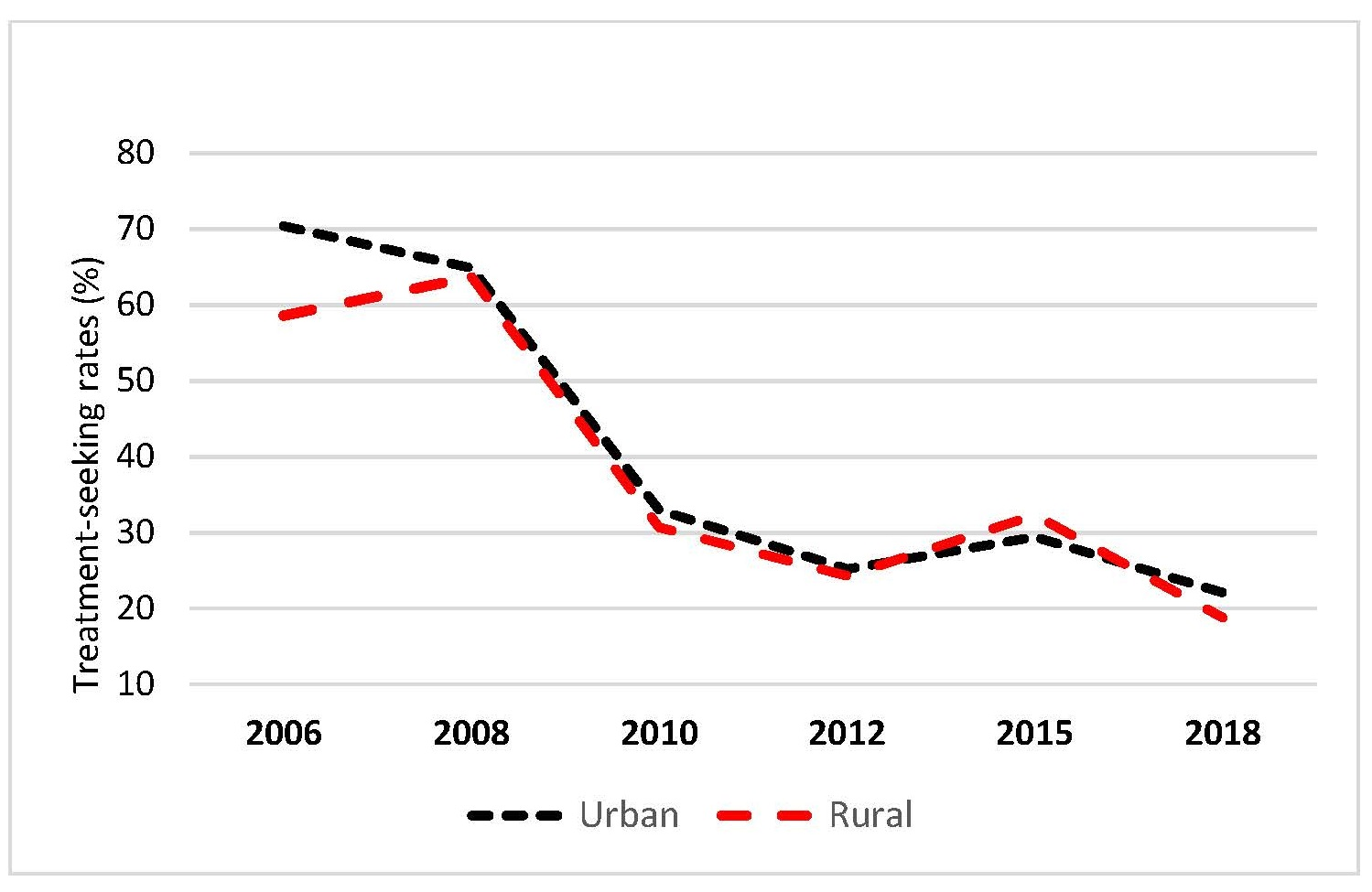

Supplement: S4 Fig — Treatment-seeking trends between rural and urban areas of Zambia during the period of study. (TIF) [file pcbi.1008669.s004.tif]
