# Supplementary material for: Modelling of malaria risk, rates, and trends: A spatiotemporal approach for identifying and targeting sub-national areas of high and low burden
Source: PLoS Comput Biol. 2021 Mar 1;17(3):e1008669. doi: 10.1371/journal.pcbi.1008669 (PMC7951982; doi:10.1371/journal.pcbi.1008669)
Supplement: S1 Appendix — (DOCX) [file pcbi.1008669.s006.docx]

**S1 APPENDIX**

Supplement to: **Modelling of Malaria Risk, Rates, and Trends: A Spatiotemporal approach for identifying and targeting sub-national Areas of High and Low Burden**

*Jailos Lubinda, Yaxin Bi, Busiku Hamainza, Ubydul Haque, Adrian J. Moore*

Section 1: Describing and explaining HMIS dataset used Data Quality and Adjustments needed

Section 2: Structure of the Bayesian Hierarchical models used

Section 3: Generating malaria risk and rates

Section 4: Working the Matrix Details

Section 5: The definition of terms used in the paper

**Section 1: Describing and explaining HMIS dataset used,** **data quality and adjustments needed**

Zambia has had a rich and unique HMIS data source since the beginning of this century. Disease information from records collected through districts, and aggregated from health facilities records have been more or less complete since 2000 [1]. During this period, a malaria case was defined as a "fever with parasites" which generally defines all those needing treatment using antimalarial drugs. The reports consist of outpatients and inpatients' data, treated based on their clinical symptoms (suspected) and on laboratory tests carried out using RDTs, and slide positivity (confirmed).

**Malaria case and mortality definitions**

Malaria mortality refers to the direct consequences of malaria infection, which primarily includes the death from a progression of mild and severe disease to death [2,3]. From a clinical view, a simplified sequence from the point of a plasmodium-infected bite is as follows [4]:

$$\boldsymbol{Infection\to asymptomatic parasitaemia\to uncomplicated illness\to severe malaria\to death}$$

This is what is generally measured by the health system, but is often hugely underestimated [2,3,5] because the true burden depends on several other factors such as transmission intensity, age, acquisition of immunity, parity, co-morbidities, and health system factors such as access and quality of health care. Hence, while Verbal Autopsy remains the primary diagnostic method of confirmation, it has poor specificity for malaria because malaria can simultaneously be both contributory and an underlying cause of death. Confirmatory accuracy still depends on many other factors within [6,7] and outside [3,8,9] the (quality of) health care system.

From 2001, all patients seeking care in the public sector due to fever could receive a malaria diagnostic test free of charge, as per health policy guidelines. A microscopy blood slide was the main malaria test available in most health facilities between 2000 and 2008. However, as per WHO guidelines, children from most sub-Saharan Africa aged below five years received treatment for all fever cases without parasite conﬁrmation [1]. Thus, due to the shortage of medical personnel and the high volume of suspected malaria cases in most public facilities, diagnosis by clinical symptoms remained a large part of the malaria diagnosis process.

Routine surveillance records have been the most abundant source of information on malaria control effects in endemic countries and worldwide. The records of malaria cases and deaths submitted to national programs and the WHO vary in quality, and most lack completeness. Hence, using the data as received is always deemed severely biased through underreporting issues due to record incompleteness or over-reporting due to the combined presumed malaria cases treated without confirmatory testing. Although routinely collected records have significantly improved their data quality in recent years, these data still need to be adjusted for various factors to reflect a more accurate picture of the malaria epidemiology [1,10].

In order to capture a more accurate picture of national malaria cases or deaths, adjustments are made on the reported country cases. The WHO's working of routinely collected data in individual countries includes adjusting malaria cases for report completeness, care-seeking rates, and parasite positivity rates (the likelihood that cases were parasite-positive). Historically though, while this was the most accurate and objective method, it was only applied in a few African countries that had enough quality data [1].

In our Zambian dataset, a lack of appropriate sub-national scale data meant that all three-adjustment parameters could not be accommodated between 2000 and 2008.

**Reporting Completeness and changing number of health facilities:** We obtained annual mean national reporting completeness from the WHO's World Malaria Reports of 2008 and 2016 [1,11]; extracted and estimated the mean health-seeking rates from three Demographic Health Surveys (DHSs) of 2001-2, 2007, and 2013-14 [12–14] [(*See Treatment seeking studies in Zambia*)](#treatment_seeeking) and used those to make completeness adjustments for 2000 to 2008. We further investigated the correlation between *reporting levels at the health facility* and *incidence rates by district;* and the *number of reporting units* and *incidence rates* during the period 2009-2015. Using a Pearson correlation matrix, the results showed that report completeness (reporting levels), as would be expected, returned a small but significant positive correlation (0.22). In contrast, there was also a small but significant negative correlation between malaria incidence and the number of health facilities (reporting units) [-0.16] and a similarly small but significant negative correlation with the actual number of reports received [-0.11].

Using a simple multiple linear regression model, only report completeness returned a significant result (95% CI. 4.8 - 12.2). These results tend to support our argument in the paper that an increasing number of health facilities did not have a significant effect in increasing patient treatment-seeking behaviour or incidence rates within districts as the rationale to add new facilities depends mostly on population growth or distance from the nearest facility and the rates will mostly be normalised by the underlying catchment populations.

**Test Positivity rates:** Data on the slide or malaria-test positivity rates were not available pre-2009. All malaria records were reported as a single figure comprising presumed and confirmed cases and cannot be disaggregated. This type of reporting was from 2000 until 2008. The introduction of a nation-wide scale-up of RDTs as the primary diagnostic test provided data at the relevant scale to enable positivity test adjustments at the district level from 2009-2015. Therefore, although we did not incorporate test positivity and cannot confirm the exact number of expected health facility reports that contributed to the records received before 2009, we have used the reporting completeness to adjust.

**Treatment-seeking rates:** For treatment-seeking rates, District level adjustments were made using the provincial level data provided from the MIS, which was the only data available. It is recognised that there may well be within-provincial variance at the district and health facility levels, but due to the absence of such fine-scale data, we could not quantify and account for them in our models. This point has also been addressed in the discussion section as a potential limitation.

MIS data is based on province-level sample clusters and may still not be fully spatially representative of within-province districts and could more appropriately be cited at a national or provincial level. Report data from several MISs showed that some districts do not have any sample clusters taken from them. For instance, the 2006 MIS shows that a total sample of 3,000 households (0.7% of total households in the country) was obtained from 120 of 17000 standard enumeration areas (SEA). Only 58 of 72 districts had sample clusters taken from them. Nonetheless, the above argument does not undermine the value and purpose of MISs. However, it merely highlights that in the absence of the raw data usable at the district level, province-level data, though offering limited spatial resolution, may still be usable, as the case here.

We note that making these adjustments is important to show a more accurate picture of malaria infections. Not having them could well affect any subsequent observed temporal and spatial trends. Furthermore, we suggest that adjusted rates are most useful when comparing rates with other countries and are of limited everyday local use by policymakers because grass-roots decisions are primarily based on actual records of reported malaria cases or deaths. Consequently, adjusted figures often pose a challenge for any reasonable comparisons for, while the results from adjusted studies may well reflect the accurate picture, they may not return a familiar picture observed on the ground by the malaria programs especially when being applied to subnational level analyses without evidence from specific subnational studies conducted at similar scales.

The data was available only at the district level (for 2000 - 2008); hence, we did not make any corrections or health facility-level adjustments. Nonetheless, for the whole dataset, we first examined individual district counts, before testing for outliers or the presence of spurious values using Cooks distance test. Two outliers were found and corrected using the district averages from the mean value before and after the outlier. We did not initially remove any other outliers from the dataset. We then examined the dispersion from the median using simple outlier statistics such as the absolute deviation from the median using DHARMa's R package - nonparametric dispersion test via standard deviation of residuals fitted vs simulated data.

Before re-analysing the data, we adjusted the data using the equation:

[1]

$$=\left( \frac{{Cases}_{confirmed}+{(Cases}_{presumed} X{Test Positivity Rate})}{Reporting completeness} \right)/Treatment Seeking Rate$$

**Data Missingness**

We used margin plots, missing data patterns and density plots to visualise the data missingness patterns in the data. 3.4%, 2.7%, and 1.4% was missing for under-five deaths, over five deaths, and across both age groups, respectively. Only 0.1% of missing values were in malaria case reports making 5% of the combined total for missing data in the whole dataset.

We used multiple imputations to create several complete versions of the dataset and replaced missing values with plausible data values [15]. Before settling on the final imputation method to use, we applied multiple imputation methods testing the efficiency and error of the imputation algorithms from Multivariate Imputation by Chained Equations implemented in MICE R package [16], the bootstrapping and predictive mean matching (PPM) from Hmisc R package [17], and the MissForest R package [18] which is random forest-based. In each case, the results are pooled into a single point estimate plus a standard error by pooling rules (Rubin's rules"). We finally chose to use a Random Forest trained on the observed values from a matrix to estimate the missing values to impute the 5% of missing values in the data. This method yields an out-of-bag (OOB) imputation error estimate minus the need to test or do elaborate cross-validation from the missing values among malaria deaths alone. The normalised mean squared error (NMSE) of imputed missing values were 0.22 (20%), and 0.072 (7%) for missing case values and 0.094 (9%) overall for the whole dataset. The figure M1 below shows density plots of imputations.

**Figure M1: density plots of imputations**


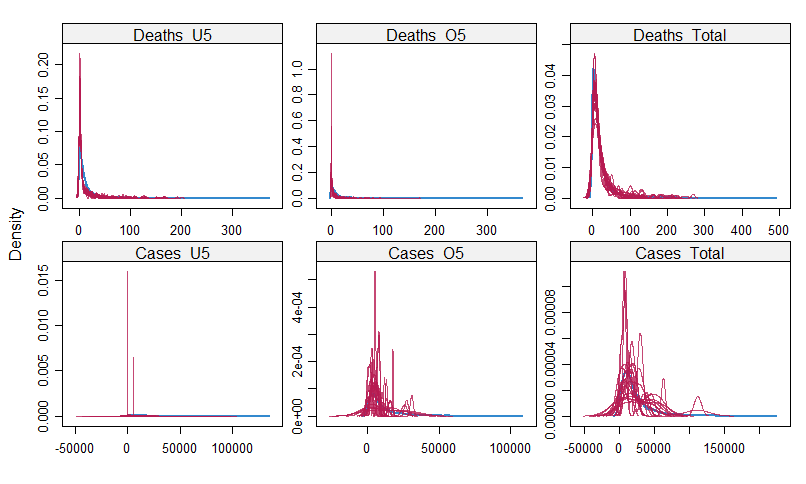


**Treatment seeking studies in Zambia**

While treatment-seeking behaviour plays a role in how many malaria/non-malaria fevers are captured at health facilities, available studies (conducted between 2007 and 2017) showed a geographical bias with up to 80% of those accessed conducted wholly or partly in Zambia's Southern Province, which is only 1 of 10 provinces. These studies are mostly geographically biased and therefore not generalisable beyond their respective study areas or sub-regions. For instance, a recent study by Edward et al. [19], conducted between 2013-2014 and 2016-2017 on care-seeking practices following the behaviour change intervention in Zambia, indicated 72.3% (N = 173) and 81.8% (N = 209) of the intervention vs comparison cohort populations sought care. However, these results were pooled (despite being across four districts) and reported only as control and intervention districts making them non-inferable elsewhere.

Other studies also conducted in southern province mostly captured high health-seeking behaviour among community-based malaria management and non-severe pneumonia in children. Over 80% (N range =174 - 362) of the cases were treated at health centres, and through CHWs [20–22] across intervention and control arms. In peri-urban settings, a case study of Lusaka showed that circa 56% (106/189) of caregivers sought care from health professionals [23]. However, it is worth noting that all these studies targeted health-seeking among young children with their caretakers' help. Information on the utilisation of health services due to fever-related illnesses by people aged 5 years and over remains limited and is mostly extrapolated from these studies to all age groups with the assumption of homogeneity in behaviour. Given this assumption and as traditionally applied by the WHO, we extracted treatment-seeking information from DHS and MIS (available at the national and provincial level) reports and used those as explained in earlier sections.

**Underestimations due to unknown subclinical malaria**

Asymptomatic individuals constitute a significant malaria reservoir of infections leading to sustained transmissions. Many studies show that subclinical or asymptomatic malaria increases as the proportion of malaria cases among febrile illness declines, and transmission continues among people with higher subclinical infections [24–27]. Subclinical is higher in low transmission areas than in high transmission areas [25,27]. We did not have information on the rates of subclinical malaria, which infers a systematic underestimation of malaria. Our approach can inform focused investigations in low-transmission settings as part of active case detection to test defined populations without prior screening for symptoms. The presence of unaccounted for and uncaptured asymptomatic malaria cases, as shown above, means very heterogeneous but significant underestimations of the actual malaria burden in the study areas and could potentially have led to different conclusions than those reported in this article because of such inherent bias.

**Section 2: Structure of the Bayesian Hierarchical models used**

The study area has 72 districts denoted by *k* = 1*, . . . ,* K, with non-overlapping districts denoted by *S* = {*S*_1_*, . . . , S_K_*}. The available data aggregated at quarterly time periods are subscripted with *t* = 1*, . . . , N*. Thus, the data are available for *K* rows (Districts) and *N* columns (Quarters). Response variables are denoted by **Y** = (*Y*_1_*, . . . , Y_N_*) *K*×*N*, where **Y***_t_* = (*Y*_1_*_t_, . . . , Y_Kt_*) represents the *K* × 1 observed vector for *K* district units at period *t*. O = indicates the vector of selected offsets (O_1_*, . . . ,* O*_N_*) *_K_*_×_*_N_*, where likewise O*_t_* = (O_1_*_t_, . . . ,*  O*_Kt_*) denotes *K* × 1 column vector of offsets for the period *t*. A vector of known covariates is denoted through, **x***_kt_* = (*x_kt_*_1_*, . . . , x_ktp_*) for district *k and* period *t*. The model takes the following structure:

*Y_kt_|μ_kt_ ~ f(*$\mathcal{y}$*_kt_ | μ_kt_,v^2^)* where *k =* 1*, . . . ,K, t =* 1*, . . . ,N,*

*g(μ_kt_) = x^T^_kt_β + O_kt_ + ѱ_kt_,*

[2]

*β~N(μᵦ,∑ᵦ)*

The specific model used implements an exact Poisson specification as:

*Y_kt_ ~* Poisson (*_μkt_*) and ln(*μ_kt_*) = *x^T^_kt_β +* *O_kt_* + *ѱ_kt_*.

[3]

**Model Specification and Structure**

We use a specialised Gaussian Markov random field (GMRF) of conditional autoregressive (CAR) structure. The spatiotemporal models represent the neighbourhood of the districts through an adjacency matrix W so that w_jr_ expresses whether districts (j, r) are spatially contiguous to produce a binary value interpreted as spatial closeness, where (w_jr_ = 1), means districts share boundaries and when (w_jr_ = 0) represents nonexistence of a shared boundary. We used the CARBayesST R package [2] to fit multiple models using data likelihood for district j and time point i is *Y*_ji_ ~Poisson (e_ji_θ_ji_) where (*Y*_ji_, e_ji_) is the observed and expected respectively.

**Capturing spatio-temporal random effects**

The model incorporates a spatio-temporal autocorrelation into the response variable **Y** through latent random effects, using CAR-type prior distributions and spatio-temporal extensions. The symmetric non-negative *K* × *K* neighbourhood controls the spatial relationship through the adjacency matrix **W**= (*w_kj_*). *W_kj_* characterises the closeness between spatial units (S*_k_,* S*_j_*). The weighted matrix creates higher values for area units with spatial adjacency, but lower or 0 values for areas spatially distant. The matrix **W** creates a binary, (*w_kj_* = 1 if spatial units (S*_k_,* S*_j_*) share a common boundary/edge and *w_kj_* = 0 if not. However, this binary specification of **W** has to fulfil three conditions: symmetry, non-negative, and row sums greater than zero. This model treats spatially proximate areal units as spatially autocorrelated while those more distant as conditionally independent.

In order to estimate the evolution of the spatial response surface over time without forcing it to be the same for each time period, the mean response with a single set of spatially and temporally autocorrelated random effects (as seen in Figure 2b and 2d) was used. Temporal autocorrelation is in turn induced through mean ρT𝜙*_t_*_−1_, while variance$\boldsymbol{Q}(\boldsymbol{W},\rho s)^{-1})$ induces the spatial autocorrelation according to the CAR equations used in the models explained earlier. Equation 4 gives the matrix:

$\left( \boldsymbol{W},\rho s \right)= \rho s\left[ \mathrm{diag}\left( \boldsymbol{W}\boldsymbol{1} \right)\boldsymbol{- W} \right]\boldsymbol{+}\left( \boldsymbol{1-}\rho s \right)\mathbf{I}$ [4]

In equation 5, 1 represents the K × 1 vector of 1's from the binary and the K × K identity matrix is denoted by I. While random effects are zero-mean centred, specific flat priors$(\rho s, \rho\mathcal{T}$) and conjugate priors $\mathcal{(T}^{2})$are given and default values (a = 1, b = 0.01) for the latter. We also tested for the collective temporal autocorrelation across all time points in the data using the Durbin-Watson test. We implemented the Cochrane-Orcutt estimation method to solve the first-order autocorrelation problem. Each of the models implemented deals with autocorrelation in specific terms, but all outputs were also subjected to the two independent autocorrelation tests to confirm the models dealt with it.

We used the Deviance Information Criterion (DIC) [28], Watanabe Akaike Information Criterion (WAIC), and its associated log pseudo-marginal likelihood (PML) to initially select the best models to use with our data from among the list of conditional autoregressive models available to us [29]. Models with similar structures were prioritised with a final choice made by the DIC and suitability to our study's objectives.

The trend reported in this study may be counter-intuitive. The combination of adjusted clinical cases with confirmed malaria cases and the continuous reduction in clinical malaria [30] due to improved availability of diagnostic tools leading to the subsequent removal of other malaria-like fevers should be captured as a reduction in malaria incidence. However, our study reports an increasing trend with a potential for underestimations, eliminating the probability of these trends being spurious.

**Cluster Trends model**

The models run did not include any covariates with the objective being to show malaria clusters among districts that shared common malaria risk trends. We assessed malaria risk clustering based on district’s shared shape-constrained temporal trends, which allowed us to test for heterogeneity vs homogeneity in temporal and spatial trends of malaria between two age groups in Zambia over the 16 year study period.

The chosen model has the capability of identifying clusters of contiguous areal units that exhibit either an elevated or reduced risk of disease compared with neighbouring areas [31,32].

In addition to the model structure described above, general model cluster trends are given by:

[5]

$$Y_{kt}\sim p\left( Y_{kt} | \mu_{kt} \right), \mathrm{where} K=1,\ldots,K,t=1,\ldots,N,$$

$$g\left( \mu_{kt} \right)=O_{kt}+{X^{T}}_{kt}\beta+\phi\sum_{s=1}^{s} \omega_{ks}f_{s}(t|\gamma)$$

Supplementary *Figure S1* shows the estimated temporal trends and 95% intervals on the risk scale, namely:

[6]

$$\theta_{t}=\exp\left( \beta_{1}+f_{s}\left( t | \gamma_{s} \right) \right)$$

Equation 7 indicates the constituents of estimates i) under five ii) over five, and iii) population-wide trend models in Figure S1. Model outputs in Figure S1a (i, ii, iii) show the lines fitting well at the 95% credible interval, with just under half (44%) of the districts (Table 1) allocated to a constant trend signifying no change in the risk of under-fives malaria mortality over time. The remainder is shared (29%, 26% - under five and 31%, 23% - over five) between the increasing and decreasing risk trends, respectively. Surprisingly, the number of no change districts increases to 50% when we combine the two age groups, signifying that not all districts exhibited the same trend between both age groups as confirmed by the trend maps (Figures 5 A(i), A(ii), B(i) and B(ii)), exhibit similar levels of spatial variation.

The trend equation formula is:

$\psi= \phi_{kt}+ \sum_{s=1}^{S} \omega_{\kappa s} f_{s}\left( t | \gamma_{s} \right),$

$\phi_{t}|\phi_{t-1}\sim N\left( \frac{\rho\sum_{j=1}^{K} \omega kj\phi}{\rho\sum_{j=1}^{K} \omega kj+1-\rho} , \frac{\mathcal{T}^{2}}{\rho\sum_{j=1}^{K} \omega kj+1-\rho} \right),$

$\mathcal{T}^{2} \sim Inverse-Gamma\left( a,b \right),$

[7]

$$\rho s, \rho\mathcal{T \sim}\mathrm{Uniform}\left( 0,1 \right).$$

$$\omega k=\left( \omega k1,\ldots, \omega kS \right) \sim\mathrm{Multinomial}\left( 1;\boldsymbol{\lambda} \right),$$

$\boldsymbol{\lambda=(}\lambda_{1},\ldots,\lambda_{S}) \sim\mathrm{Dirichlet}\left( \boldsymbol{\alpha}=\left( \alpha_{1},\ldots,\alpha_{S} \right) \right),$

where ϕ−*k* = (*ϕ*_1_*, . . . , ϕ_k_*−1*, ϕ_k_*+1*, . . . , ϕ_K_* ).

### Areas are initially clustered according to their temporal trends; then we use global probabilities to associate candidate trends in the output and trend interpretation. The model also visualised the classifications assigned to trends using maximum posterior probabilities of certainty thresholds (0.33 – 0.5, 0.5 -0.75, 0.75 - 1). The trend function used for the model and its interpretation can be accessed elsewhere [33]*.*

We also tested monotonic trend alternatives before selecting the one used. Our original choice and rationale as we tested the monotonic alternatives (not included or discussed here) were guided by the understanding that the number of knots controls the estimated trend's wiggliness [33]. Ruppert et al. [34] discuss that if one uses a linear spline with enough knots, increasing the number of knots has no appreciable effect on a penalised fit, then increasing the degree of the spline is also unlikely to have a noticeable effect. Claeskens et al. [35] further refined the justification to prove that a smaller number of knots leads to a smaller averaged mean squared error. Hence, given the small number of time points, Q = 2 was enough, and any more would not have much effect, although we believe fewer would lead to larger MSE. However, our final choice model was not monotonic and did not need any knots.

**Section 3: Generating malaria risk and rates**

**Relative Risk**

# We calculated indirectly Standardised Mortality Ratio (SMR) and Standardised Incidence Ratios (SIR). The SIR/SMR are ratios between observed counts of deaths/incidence in a study population and the expected deaths/incidence rates, depending on the age-specific rates in a standard population. These ratios are dependent on the demographic size and profile of the study population. High risk is determined when the ratio of observed/expected counts is greater than 1.0 in the study population, while low risk is when the ratio is less than 1.0. In order to make three classes *low, medium,* and *high* that would conform to the model classification for risk trends, we classified all below 1.0 as low, those between 1.0 – 1.5 as medium (equivalent to 50% increase above national population) and all over 1.5 as *high.*

**Malaria Rates**

Malaria mortality rates were calculated as death counts per 10,000 population of the specific age group, while incidence rates were calculated per 1,000. For comparability between risks and trends, we scaled all rates to a range of between 0 and 1. We then classified the values into three groupings of 0 - 0.33 = *low*, 0.34 - 0.67 = *medium*, and 0.68 – 1 = *high*.

**Section 4: Working the composite Matrix for visualising *High/Low* burden areas**

We created a matrix composed of the district results from the models for trend clusters, rates, and risk. ***Incidence and mortality rate:*** in the output table *(see supplementary table S1)* and as earlier described, we classified mortality and incidence in the range of 1 - 3 where high = 3, medium = 2, and low = 1.

For ***relative risk*** (RR), <1 =“*low risk*”, 2 = “*medium risk*” with RR between 1-1.5 (denoting 0.1-50% risk higher risk), while 3 = *“high risk”* with RR more than 50% - 200% higher than the national average. For the ***trend classification*** by the model, we denote *decreasing trend = 1*, no*-change* (*constant*) *= 2* and *Increasing trend = 3* which inherently meant the equivalents of low, medium and high, respectively. Specifically, we chose a rating scale of high = 3, medium = 2, and low = 1 in all three criteria.

In the *final mapping* of *high/low* burden areas*,* a similar method was implemented resulting in a logical classification approach recognising a high combined matrix score to represent an area of *high* *malaria* *burden* and consequently an area of high potential impact to effective interventions. The opposite was also true denoting low matrix scores as areas suitable for malaria elimination. We then imported this dataset into ArcGIS 10.5, where each combined district score was derived from a product of scores across risk, rates, and trend from the matrix. For example, a district with "high RR" (weight 3), "Increasing trend" (weight 3), and a high rate (weight 3) would give a product score of 27 (3 x 3 x 3), while having one of the scores as 2 would make the final score =18 (3 x 2 x 3). We then used manually defined classes to make the class cut-offs, as shown in the matrix t*able S1* to visualise the results. Here, any weighting scores could also be applied to denote the particular metric's importance.

Although scores were multiplied across the confusion matrix columns to obtain a total score for each district out of preference for easy mapping of classes, we also explored other ways such as addition, averaging, and rescaling to aggregate our matrix scores in the most understandable way. The latter methods did not change the final visualisation result but made class exclusivity more challenging to achieve because they generated very close scores. Product scores, however, gave the best classes. We decided to apply the matrix for under-five and over-five age group incidence or mortality separately (same as mortality) as combining them concealed the matrix's precise age-specific constructs.

**Section 5: The definition of terms used in the paper**

WHO: World Health Organization

GTS: Global Technical Strategy

IRS: Indoor Residual Spraying

GIS: Geographic Information System

MIS: Malaria Information System

ACT: Artemisinin-based Combination Therapy

RDT: Rapid Diagnostic Test

E8: Elimination 8 Malaria Initiative

Under-fives: Children aged under five years old

Over-fives: People aged over five years old

WMR: World Malaria Report

**REFERENCES**

1. World Health Organization. World Malaria Report 2008. World Health Organization: Geneva; 2008.

2. Greenwood BM, Bradley AK, Greenwood AM, Byass P, Jammeh K, Marsh K, et al. Mortality and morbidity from malaria among children in a rural area of The Gambia, West Africa. Trans R Soc Trop Med Hyg. Elsevier; 1987;81:478–86.

3. Mudenda SS, Kamocha S, Mswia R, Conkling M, Sikanyiti P, Potter D, et al. Feasibility of using a World Health Organization-standard methodology for Sample Vital Registration with Verbal Autopsy (SAVVY) to report leading causes of death in Zambia: results of a pilot in four provinces, 2010. Popul Health Metr. Springer; 2011;9:40.

4. World Health Organization. Severe malaria. Trop Med Int Heal. WILEY-BLACKWELL 111 RIVER ST, HOBOKEN 07030-5774, NJ USA; 2014;19:7–131.

5. Snow R., Winstanley M., Marsh V., Newton CRJ., Waruiru C, Mwangi I, et al. Childhood deaths in Africa: uses and limitations of verbal autopsies. Lancet. Elsevier; 1992;340:351–5.

6. Reyburn H, Mbatia R, Drakeley C, Carneiro I, Mwakasungula E, Mwerinde O, et al. Overdiagnosis of malaria in patients with severe febrile illness in Tanzania: a prospective study. BMJ. British Medical Journal Publishing Group; 2004;329:1212.

7. Taylor TE, Fu WJ, Carr RA, Whitten RO, Mueller JG, Fosiko NG, et al. Differentiating the pathologies of cerebral malaria by postmortem parasite counts. Nat Med. Nature Publishing Group; 2004;10:143–5.

8. Lynch M, Korenromp E, Eisele T. New global estimates of malaria deaths (vol 380, pg 559, 2012). Lancet. ELSEVIER SCIENCE INC 360 PARK AVE SOUTH, NEW YORK, NY 10010-1710 USA; 2012;380:1148.

9. White NJ, Dondorp AM, Faiz A, Mishra S, Hien TT. New global estimates of malaria deaths. Lancet. Elsevier; 2012;380:559–60.

10. Ashton RA, Bennett A, Yukich J, Bhattarai A, Keating J, Eisele TP. Methodological considerations for use of routine health information system data to evaluate malaria program impact in an era of declining malaria transmission. Am J Trop Med Hyg. 2017;97:46–57.

11. World Health Organization. World Malaria Report 2016. World Health Organization: Geneva; 2016.

12. Central Statistical Office, Central Board of Health and OM. Zambia Demographic Health Survey 2001-2002 [Internet]. Measuredhs.Com. Calverton, Maryland, USA; 2002. Available from: http://www.measuredhs.com/pubs/pdf/FR136/FR136.pdf%5Cnpapers2://publication/uuid/01C995EB-49F4-4D2E-8B61-14F905895615%5Cnpapers2://publication/uuid/2EB4C494-E2A1-410C-88FB-CE1102E9A4F0

13. Central Statistical Office M of H, Centre TDR, Zambia U of, Inc., MI. Zambia Demographic and Health Survey 2007. Calverton, Maryland, USA; 2009.

14. Central Statistical Office, Ministry of Health and II. Zambia Demographic Health Survey 2013-14. Stud. Fam. Plann. Rockville, Maryland, USA; 2014.

15. Van Buuren S. Flexible imputation of missing data. Chapman and Hall/CRC; 2018.

16. Buuren S van, Groothuis-Oudshoorn K. mice: Multivariate imputation by chained equations in R. J Stat Softw. University of California, Los Angeles; 2010;1–68.

17. Harrell Jr FE, Harrell Jr MFE. Package ‘Hmisc.’ CRAN2018. 2019;235–6.

18. Stekhoven DJ, Bühlmann P. MissForest—nonparametric missing value imputation for mixed-type data. Bioinformatics. Oxford University Press; 2011;28:112–8.

19. Edward A, Sanchez JF, Chhorvann C, Bowles C, Malama S, Chege J. Impact of community oriented interventions on pediatric care-seeking practices-A multi-country study in Cambodia, Guatemala, Kenya And Zambia. mortality [Internet]. 2018;6:8. Available from: https://www.oatext.com/impact-of-community-oriented-interventions-on-pediatric-care-seeking-practices-–-a-multi-country-study-in-cambodia-guatemala-kenya-and-zambia.php#Article_Info

20. Yeboah-Antwi K, Pilingana P, Macleod WB, Semrau K, Siazeele K, Kalesha P, et al. Community case management of fever due to malaria and pneumonia in children under five in Zambia: a cluster randomised controlled trial. PLoS Med. Public Library of Science; 2010;7:e1000340.

21. Seidenberg PD, Hamer DH, Iyer H, Pilingana P, Siazeele K, Hamainza B, et al. Impact of integrated community case management on health-seeking behavior in rural Zambia. Am J Trop Med Hyg [Internet]. The American Society of Tropical Medicine and Hygiene; 2012;87:105–10. Available from: https://www.ncbi.nlm.nih.gov/pubmed/23136285

22. Hamooya BM, Chongwe G, Dambe R, Halwiindi H. Treatment-seeking behaviour for childhood fever among caretakers of Chivuna and Magoye rural communities of Mazabuka District, Zambia: a longitudinal study. BMC Public Health. BioMed Central; 2016;16:762.

23. Sasaki S, Fujino Y, Igarashi K, Tanabe N, Muleya CM, Suzuki H. Access to a health facility and care‐seeking for danger signs in children: before and after a community‐based intervention in Lusaka, Zambia. Trop Med Int Heal. Wiley Online Library; 2010;15:312–20.

24. Harris I, Sharrock WW, Bain LM, Gray K-A, Bobogare A, Boaz L, et al. A large proportion of asymptomatic Plasmodium infections with low and sub-microscopic parasite densities in the low transmission setting of Temotu Province, Solomon Islands: challenges for malaria diagnostics in an elimination setting. Malar J. BioMed Central; 2010;9:254.

25. Okell LC, Bousema T, Griffin JT, Ouédraogo AL, Ghani AC, Drakeley CJ. Factors determining the occurrence of submicroscopic malaria infections and their relevance for control. Nat Commun. Nature Publishing Group; 2012;3:1237.

26. Björkman AB. Asymptomatic low-density malaria infections: a parasite survival strategy? Lancet Infect Dis. Elsevier; 2018;18:485–6.

27. Mangeni JN, Obala A, Ongore D, Mwangi A, O’Meara WP, J.N. M, et al. Prevalence of asymptomatic malaria infections and associated risk factors in a high transmission region in Western Kenya. Am J Trop Med Hyg [Internet]. 2016;95:290. Available from: http://www.ajtmh.org/deliver/fulltext/14761645/95/5_Suppl/ASTMH16AbstractBook.pdf?itemId=/content/journals/10.4269/ajtmh.abstract2016&mimeType=pdf&containerItemId=content/journals/14761645

28. Spiegelhalter DJ, Best NG, Carlin BP, Van Der Linde A. Bayesian measures of model complexity and fit. J R Stat Soc Ser B (Statistical Methodol. Wiley Online Library; 2002;64:583–639.

29. Lee D, Rushworth A, Napier G. Spatio-Temporal Areal Unit Modeling in R with Conditional Autoregressive Priors Using the CARBayesST Package. J Stat Softw [Internet]. 2018;84. Available from: http://www.jstatsoft.org/v84/i09/

30. President’s Malaria Initiative. Malaria Operational Plan FY 2018. 2018;

31. Charras-Garrido M, Abrial D, Goër J De, Dachian S, Peyrard N. Classification method for disease risk mapping based on discrete hidden Markov random fields. Biostatistics [Internet]. 2012;13:241–55. Available from: http://dx.doi.org/10.1093/biostatistics/kxr043

32. Anderson C, Lee D, Dean N. Identifying clusters in Bayesian disease mapping. Biostatistics [Internet]. 2014;15:457–69. Available from: http://dx.doi.org/10.1093/biostatistics/kxu005

33. Napier G, Lee D, Robertson C, Lawson A. A Bayesian space-time model for clustering areal units based on their disease trends. Biostatistics [Internet]. 2018;00:1–17. Available from: https://academic.oup.com/biostatistics/advance-article-abstract/doi/10.1093/biostatistics/kxy024/5039880

34. Ruppert D, Wand MP, Carroll RJ. Semiparametric regression. Cambridge university press; 2003.

35. Claeskens G, Krivobokova T, Opsomer JD. Asymptotic properties of penalised spline estimators. Biometrika. Oxford University Press; 2009;96:529–44.
